# Supplementary material for: Single nucleus multi-omics regulatory landscape of the murine pituitary
Source: Nat Commun. 2021 May 11;12:2677. doi: 10.1038/s41467-021-22859-w (PMC8113460; doi:10.1038/s41467-021-22859-w)
Supplement: Supplementary file 1 — Supplementary Information [file 41467_2021_22859_MOESM1_ESM.pdf]

Supplementary Information

Ruf-Zamojski et al. Single nucleus multi-omics regulatory landscape of the murine pituitary

SUPPLEMENTARY TABLES

Supplementary Table 1: Summary sample table

a. All Samples

Sample #	Sample name	Dissociation type	Material used	Assay
1	Male mouse pit #2	standard	cells	SC RNAseq v2
2	Male mouse pit #1	standard	cells	SC RNAseq v2
3	Male mouse pit #5	standard	cells	SC RNAseq v3
4	Male mouse pit #6	standard	cells	SC RNAseq v3
5	Female mouse pit #1	gentle	cells	SC RNAseq v3
6	Female mouse pit #1	gentle	nuclei	SN RNAseq v3
7	Male mouse pit #4	standard	cells	SC RNAseq v3
8	Male mouse pit #4	standard	nuclei	SN RNAseq v3
9	Female mouse pit #2	gentle	cells	SC RNAseq v3
10	Female mouse pit #2	gentle	nuclei	SN RNAseq v3
11	Female mouse pit #1	gentle	nuclei	SC ATAC, v1
12	Male mouse pit #4	standard	nuclei	SC ATAC, v1
13	Female mouse pit #2	gentle	nuclei	SC ATAC, v1
14	Male pit#1	none, snap-frozen	nuclei	SN RNAseq v3
15	Male pit#1	none, snap-frozen	nuclei	SC ATAC, v1
16	Male pit#2	none, snap-frozen	nuclei	SN RNAseq v3
17	Male pit#2	none, snap-frozen	nuclei	SC ATAC, v1
18	Male pit#3	none, snap-frozen	nuclei	SN RNAseq v3
19	Male pit#3	none, snap-frozen	nuclei	SC ATAC, v1
20	Female pit#1	none, snap-frozen	nuclei	SN RNAseq v3
21	Female pit#1	none, snap-frozen	nuclei	SC ATAC, v1
22	Female pit#2	none, snap-frozen	nuclei	SN RNAseq v3
23	Female pit#2	none, snap-frozen	nuclei	SC ATAC, v1
24	Female pit#3	none, snap-frozen	nuclei	SN RNAseq v3
25	Female pit#3	none, snap-frozen	nuclei	SC ATAC, v1
26	30 pooled male pituitarie	none, snap-frozen	nuclei	SnmC seq2

**Total cells SC RNAseq**	**31959**	cells	from 7 individual animals (5 males and 2 females)
**Total cells SN RNAseq**	**83879**	nuclei	from 9 individual animals (5 females and 4 males)
**Total cells ATAC seq**	**54181**	nuclei	from 9 individual animals (5 females and 4 males)
**Total cells methylation**	**2756**	nuclei	from 30 pooled animals (all males)

b. sc RNAseq metrics

Sample #	Sample name	Number of cells	Mean reads/cell	Median genes/cell	Number of reads	Valid barcodes	median UMI per cell	Sequencing saturation	Reads map to genome	Reads map to transcriptome	total genes detected
1	Male mouse pit #2	1,691	231,650	1,341	391,721,124	97.10%	3,942	95.40%	93.60%	71.70%	17,715
2	Male mouse pit #1	542	619,209	879	335,611,576	97.10%	2,684	97.00%	93.40%	74.10%	14,787
3	Male mouse pit #5	3,037	134,016	2,618	407,008,052	97.40%	7,661	96.20%	94.00%	78.20%	20,204
4	Male mouse pit #6	2,476	174,900	2,279	433,052,748	97.40%	6,275	93.70%	95.60%	76.60%	19,461
5	Female mouse pit #1	11,974	31,577	2,294	378,107,244	97.90%	6,408	57.50%	93.90%	70.50%	22,129
6	Female mouse pit #1	28,109	15,474	1,036	434,978,626	97.00%	3,180	37.90%	93.30%	59%	22,336
7	Male mouse pit #4	2,565	160,128	1,903	410,730,198	97.10%	4,394	86.20%	74.60%	45.20%	20,433
8	Male mouse pit #4	9,901	45,280	1,022	448,323,379	96.70%	2,880	70.40%	91.50%	52%	21,600
9	Female mouse pit #2	9,674	45,283	2,458	438,074,966	97.30%	6,512	66.60%	88.90%	66.50%	21,928
10	Female mouse pit #2	10,162	38,205	1,129	388,244,083	96.60%	3,789	62.50%	93.70%	60.80%	21,131

c. sc ATACseq metrics

Sample #	Sample name	Number of cells	Median fragments/cell	Fraction of fragments overlapping any targeted region	Fraction of transposition events peaks peaks in cell barcode	Total number of read pairs	Fraction of reads with a valid barcode	Enrichment score of transcription start sites	Percent duplicates	Fraction of fragments overlapping called peaks
11	Female mouse pit #1	10,420	14,342	75%	70.20%	806,436,343	94.90%	7.7	33.90%	72.20%
12	Male mouse pit #4	2,536	13,044	55.70%	25.90%	629,110,764	95.10%	3.08	2.70%	27.70%
13	Female mouse pit #2	7,782	14,150	66.40%	37.20%	713,242,457	94.60%	6.92	23.80%	58.70%

a. Table showing all the samples run in the study, along with the dissociation and type of assay performed. b. sc RNAseq metrics for all samples except snap-frozen pituitaries. c. sc ATACseq metrics for all samples except snap-frozen pituitaries.

Supplementary Table 2: Metrics obtained for each of the six snap-frozen pituitary samples for the sn assays.

a. snRNAseq metrics

	Female #1	Female #2	Female #3	Male #1	Male #2	Male #3	Average
Number of cells	7,406	5,725	4,515	7,322	4,726	6,279	5,996
Mean reads/cell	26,738	37,757	45,381	29,171	43,339	34,301	36,115
Median genes/cell	1,934	2,374	1,886	1,791	1,903	1,784	1,945
Number of reads	198,024,328	216,159,707	204,894,708	213,593,436	204,818,325	215,374,733	208,810,873
Valid barcodes	96%	95.90%	96.22%	95.30%	95.60%	95.60%	95.77%
Valid UMI	100%	100%	100%	100%	99.90%	99.90%	99.97%
Sequencing saturation	39.60%	49.20%	67.30%	55.60%	67%	66.50%	57.53%
Reads map to genome	89.80%	90.50%	93.10%	93.20%	94%	94.20%	92.47%
Reads map to transcriptome	46.50%	48.50%	59.10%	50.00%	52%	39.20%	49.23%
Total genes detected	23,958	23,982	22,940	23,684	23,348	23,431	23,557

b. snATACseq metrics

	Female #1	Female #2	Female #3	Male #1	Male #2	Male #3	Average
Number of cells	4,968	10,248	13,338	2,840	967	1,082	5,574
Median fragments/cell	22,722	15,329	11,740	34,043	44,182	48,446	29,410
Fraction of fragments overlapping any targeted region	74.90%	68.60%	71.60%	71.80%	76.10%	71%	72%
Fraction of transposition events in peaks in cell barcodes	73.20%	65.70%	65.80%	65.40%	75.60%	67.80%	68.92%
Total number of read pairs	342,097,953	321,522,809	345,158,674	313,157,573	318,016,006	397,789,926	339,623,824
Fraction of reads with a valid barcode	96.40%	96.10%	96.10%	96.10%	96.50%	96.40%	96.27%
Enrichment score of transcription start sites	11.36	8.26	9.58	9.92	11.82	9.08	10.00333333
Percent duplicates	44.50%	32.70%	32.20%	45%	63.40%	59.70%	46.25%
Fraction of fragments overlapping called peaks	74.70%	67.20%	67.50%	68%	77.20%	69.40%	70.58%

Supplementary Table 3: Cell type proportions identified from the merged analysis of the snap-frozen pituitary samples

a. snRNAseq

	FEMALES
**Cell types**	**%**
Corticotropes	**2.69**
Endothelial	**0.55**
Gonadotropes (1-2)	**1.82**
Gonadotropes (3)	**0.75**
Lactotropes (1-2)	**26.31**
Lactotropes (3)	**10.18**
Leukocytes	**0.23**
Macrophages	**0.21**
Melanotropes	**7.06**
Pericytes	**1.21**
Pit1- lineage	**0.41**
Pituicytes	**0.92**
Proliferating	**5.31**
Somatotropes 0	**3.64**
Somatotropes	**27.33**
Stem cells A	**5.68**
Stem cells B	**4.51**
Thyrotropes	**1.19**

	MALES
**Cell types**	**%**
Corticotropes	**3.11**
Endothelial	**0.85**
Gonadotropes	**5.56**
Lactotropes	**19.51**
Leukocytes	**0.22**
Macrophages	**0.21**
Melanotropes	**7.70**
Pericytes	**1.09**
Pit1- lineage	**0.40**
Pituicytes	**0.88**
Proliferating	**1.27**
Somatotropes 0	**6.03**
Somatotropes	**35.09**
Somatotropes (3)	**9.50**
Stem cells A	**3.29**
Stem cells B	**4.01**
Thyrotropes	**1.28**

b. snATACseq

	FEMALES
**Cell types**	**%**
Corticotropes	**4.99**
Endothelial	**0.75**
Gonadotropes (1-2)	**2.03**
Gonadotropes (3)	**2.41**
Immnune cells	**1.18**
Lactotropes (1-2)	**19.77**
Lactotropes (3)	**16.76**
Melanotropes	**6.87**
Pericytes	**1.23**
Pituicytes	**0.84**
Proliferating Lactotropes	**1.67**
Proliferating Somatotropes	**1.39**
Somatotropes (1-2)	**13.59**
Somatotropes (3)	**14.72**
Stem cells	**9.48**
Thyrotropes	**2.32**

	MALES
**Cell types**	**%**
Corticotropes	**3.63**
Endothelial	**0.82**
Gonadotropes	**5.67**
Immune cells	**0.50**
Lactotropes	**21.94**
Melanotropes	**7.27**
Pericytes	**1.15**
Pituicytes	**0.73**
Somatotropes	**44.08**
Somatotropes 0	**6.47**
Stem cells	**6.40**
Thyrotropes	**1.32**

Tables showing the percentages of cells obtained after merging the datasets by sex for the snRNAseq (**a**.) and the snATACseq (**b.**). Note that the number in parenthesis next to a cell-type corresponds to the animal(s) in which this cell-type was identified. Also refer **to Fig.4**.

Supplementary Table 4: Cell type proportions identified from the individual sn assays of the snap-frozen pituitary samples

a.

	FEMALES					MALES					Males/females
**Cell types**	**#1**	**#2**	**#3**	**Average**	**Std dev**	**#1**	**#2**	**#3**	**Average**	**Std dev**	**Stat. sign.**
lactotropes (%)	31.22	38.72	37.67	**35.87**	4.06	20.91	15.08	19.30	**18.43**	3.01	**Yes**
somatotropes (%)	27.32	27.72	36.98	**30.67**	5.47	46.31	56.97	47.17	**50.15**	5.92	**Yes**
melanotropes (%)	9.11	7.06	5.57	**7.25**	1.78	8.78	5.85	8.80	**7.81**	1.70	**No**
gonadotropes (%)	3.20	2.84	3.80	**3.28**	0.48	6.55	5.41	6.41	**6.12**	0.62	**No**
corticotropes (%)	3.48	3.22	2.03	**2.91**	0.77	4.06	1.95	3.68	**3.23**	1.12	**No**
thyrotropes (%)	1.95	1.38	1.67	**1.67**	0.28	1.47	1.42	1.74	**1.54**	0.17	**No**
stem cells (%)	12.79	10.22	8.29	**10.43**	2.26	7.33	8.66	7.92	**7.97**	0.67	**No**
proliferating (%)	6.18	5.04	2.48	**4.57**	1.89	1.13	0.55	1.48	**1.05**	0.47	**No**
Pericytes (%)	1.64	1.74	0.45	**1.28**	0.72	1.06	1.29	1.37	**1.24**	0.16	**No**
endothelial (%)	0.89	1.04	0.00	**0.64**	0.56	0.80	1.21	1.03	**1.01**	0.20	**No**
macrophages (%)	0.64	0.55	0.36	**0.52**	0.14	0.66	0.46	0.36	**0.49**	0.15	**No**
pituicytes (%)		0.47	0.69	**0.58**	0.16	0.95	1.14	0.75	**0.95**	0.20	**No**

b.

	FEMALES					MALES					Males/females
**Cell types**	**#1**	**#2**	**#3**	**Average**	**Std dev**	**#1**	**#2**	**#3**	**Average**	**Std dev**	**Stat. sign.**
lactotropes (%)	33.18	40.31	39.14	**37.54**	3.83	23.58	22.61	17.63	**21.27**	3.20	**Yes**
somatotropes (%)	25.54	27.92	32.30	**28.59**	3.43	43.21	56.48	48.22	**49.30**	6.70	**Yes**
melanotropes (%)	7.30	5.99	6.35	**6.55**	0.68	7.44	5.00	8.22	**6.89**	1.68	**No**
gonadotropes (%)	3.67	3.85	4.48	**4.00**	0.42	5.86	5.00	6.03	**5.63**	0.55	**No**
corticotropes (%)	5.12	4.11	4.82	**4.68**	0.52	4.10	3.52	3.38	**3.67**	0.38	**No**
thyrotropes (%)	2.50	1.79	2.59	**2.29**	0.43	1.62	1.14	1.19	**1.31**	0.26	**No**
stem cells (%)	10.40	8.70	9.27	**9.46**	0.86	6.90	6.25	6.85	**6.67**	0.36	**No**
pituicytes (%)	1.16	0.58	1.06	**0.93**	0.31	0.75		3.74	**2.25**	2.11	**No**
multiplets (%)	11.14	6.75		**8.94**	3.11	6.54		4.75	**5.65**	1.27	**No**

c.

	**MALES**
**Cell types**	Pooled
lactotropes (%)	20.57
somatotropes (%)	41.80
melanotropes (%)	7.44
gonadotropes (%)	4.75
corticotropes (%)	4.17
thyrotropes (%)	2.21
stem cells (%)	8.13
pericytes (%)	3.16
endithelial (%)	6.39
outliers (%)	1.38

Table showing the percentages of cells obtained after analysis by snRNAseq (**a**.), snATACseq (**b**.), and sn-methylation (**c**.).

Supplementary Table 5: Somatotrope sub-type proportions identified from the individual sn assays of the snap-frozen pituitary samples

a. snRNAseq

	FEMALES					MALES				
**Cell types**	**#1**	**#2**	**#3**	**Average**	**Std dev**	**#1**	**#2**	**#3**	**Average**	**Std dev**
Somatotropes 0 (lacto-somatotropes)	0.00	0.00	0.00	**0.00**	0.00	5.23	4.10	5.34	**4.89**	0.69
Somatotropes 1	13.88	16.43	22.24	**17.52**	4.29	20.60	27.18	22.71	**23.50**	3.36
Somatotropes 2	13.44	11.28	14.74	**13.16**	1.75	20.48	23.87	19.12	**21.15**	2.45

b. snATACseq

	FEMALES					MALES				
**Cell types**	**#1**	**#2**	**#3**	**Average**	**Std dev**	**#1**	**#2**	**#3**	**Average**	**Std dev**
Somatotropes 0 (lacto-somatotropes)	0.00	0.00	0.00	**0.00**	0.00	0.00	0.00	0.00	**0.00**	0.00
Somatotropes 1	16.22	13.94	20.99	**17.05**	3.60	22.97	31.02	21.46	**25.15**	5.14
Somatotropes 2	9.33	13.97	11.31	**11.54**	2.33	20.24	25.45	26.76	**24.15**	3.45

Tables showing the percentages of cells obtained after analysis of the individual animals by snRNAseq (**a**.) and snATACseq (**b.**). Note that Somatotropes 1 and Somatotropes 2 are part of the same main cluster, representing the poles of that cluster, refer to **Supplementary Figs. 4** and **5**.

Supplementary Table 6: Cell type proportions identified from the integrated analysis of the snap-frozen pituitary samples

	Females			Males	Males		
	1	2	3	Methylation	1	2	3
Corticotropes	4.35	3.94	4.05	0.77	4.10	2.14	3.57
Endothelial	0.75	1.04	0.00	4.60	0.69	1.22	0.96
Gonadotropes	3.36	3.28	4.07	9.71	6.36	5.23	6.45
Lactotropes	31.44	39.33	38.79	33.96	21.85	16.13	19.25
Macrophages	0.68	0.73	1.02	0.81	0.67	0.48	0.36
Melanotropes	8.69	6.66	5.92	9.35	8.47	5.72	8.87
Pericytes	1.66	1.93	0.95	3.09	0.97	1.46	1.45
Pituicytes	1.42	0.55	0.90	1.25	0.88	1.04	0.79
Proliferating	5.91	3.76	1.80	0.81	1.15	0.73	1.40
Somatotropes_0	0.00	0.00	0.00	0.44	4.74	2.96	5.02
Somatotropes_1	15.08	15.74	19.81	18.29	20.80	25.82	22.70
Somatotropes_2	12.27	11.99	11.68	5.81	20.51	27.59	19.66
Stem_cells	12.23	9.48	8.81	9.60	7.26	8.11	7.80
Thyrotropes	2.14	1.58	2.20	1.51	1.54	1.37	1.70
TOTAL	100	100	100	100	100	100	100

Indicated in the tables are the percentages of cells identified in each cluster and sub-cluster from the integrated multi-omics analysis of the individual samples.

Supplementary Table 7: SCENIC highest activity regulons per cell type

**Cell type**												
**Somatotrope 1**	Rora	Nr3c1	Zeb 1	Zbtb16	Tcf4	Nfat5	Pknox2	Cux1	Pou1f1	Pou2f1	Thrb	Tcf12
**Somatotrope 2**	Rora	Nr3c1	Zeb 1	Zbtb16	Nfat5	Tcf4	Cux1	Pou1f1	Thrb	Tcf12	Pou2f1	Smad3
**Lactotropes**	Rora	Zeb1	Thrb	Tcf4	Trps1	Pknox2	Nfat5	Nr1h4	Pou6f2	Myb	Pou1f1	Pou2f1
**Gonadotropes**	Rora	Tcf4	Zeb 1	Tcf12	Nfat5	Pitx1	Smad3	Nr3c1	Pknox2	Gata2	Pou2f1	Foxl2
**Corticotropes**	Rora	Nr3c1	Zeb1	Tcf4	Nfat5	Pknox2	Zbtb16	Pou2f1	Tcf12	Mkx	Cux1	Twist2
**Melanotropes**	Rora	Tcf4	Zeb 1	Nfat5	Mkx	Pax7	Pou2f1	Tcf12	Ascl1	Cux1	Gmeb2	Tbx19
**Thyrotropes**	Rora	Zeb1	Tcf4	Nr3c1	Nfat5	Thrb	Pknox2	Pou2f1	Tcf12	Cux1	Pitx1	Pou1f1
**Stem cells**	Nfat5	Tcf4	Rora	Rfx4	Nr3c1	Zmat4	Pax6	Pou2f1	Cpeb1	Tcf12	Tcf7l2	Tcf7l2

Supplementary Table 8: Primers used nd their sequences

**Primers**	**Sequences**
Fshb forward	5'-GAAGAGTGCCGTTTCTGCAT-3'
Fshb reverse	5'-CCGAGCTGGGTCCTTATACA-3'
Cga (*α-subunit*) forward	5'-AGCTAGGAGCCCCCATCTAC-3'
Cga (*α-subunit*) reverse	5'-TTCTCCACTCTGGCATTTCC-3'
Gapdh forward	5'-GGCATTGCTCTCAATGACAA-3'
Gapdh reverse	5'-TGTGAGGGAGATGCTCAGTG-3'
primer targeting mouse rs11031006	5'- CTGGAATTTAATATTGCTCTGCCCTGTGATATTTATTTCAAGGTTAGTAGAAATGTAGCTACCTCCTGTAATGACAAATGA-3'
rs11031006 forward	5'-TGAATGCTATTTTGTGGCAACT-3'
rs11031006 reverse	5'-TCATTTGTCATTACAGGAGGTAGC-3'

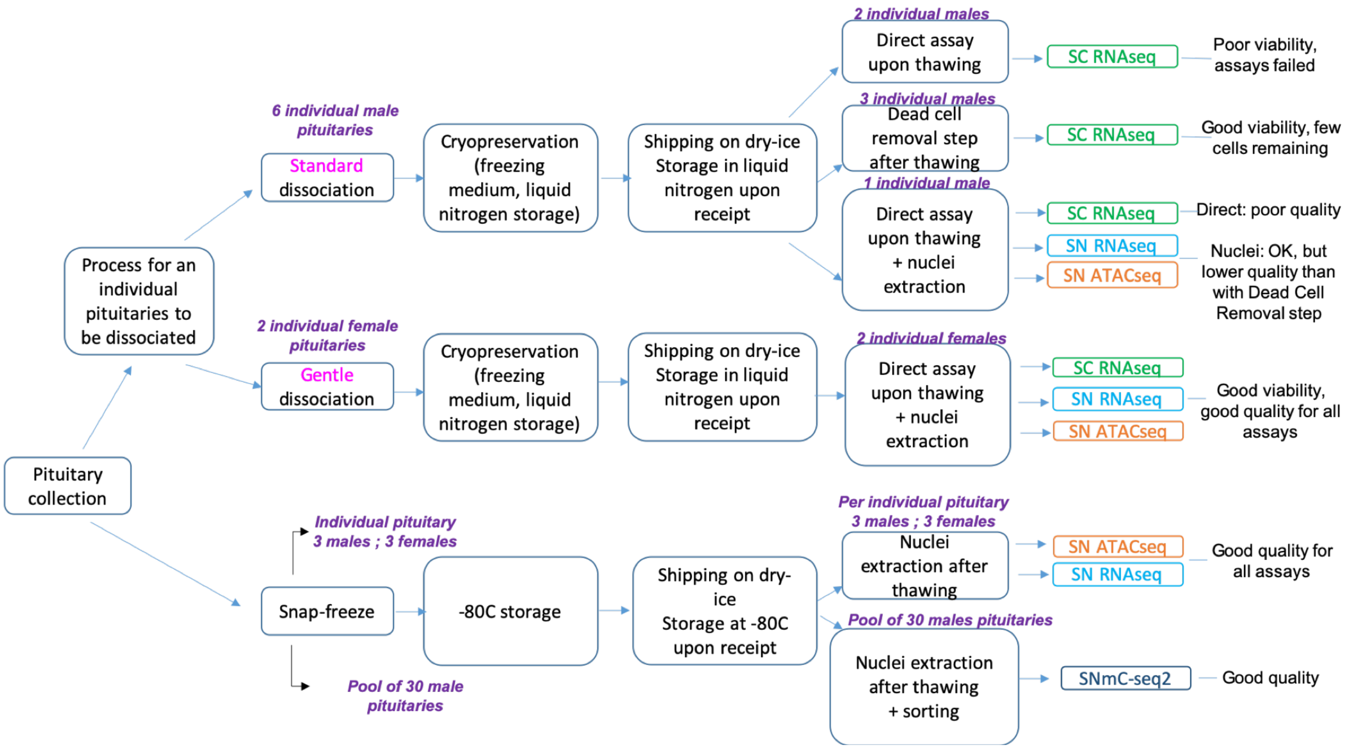

Supplementary Figure 1:
Schematic showing the different experimental strategies that were tested, from dissociated cells to whole snap-frozen pituitaries, standard to gentle cell dispersion, and the sc assays that were performed and their outcomes.

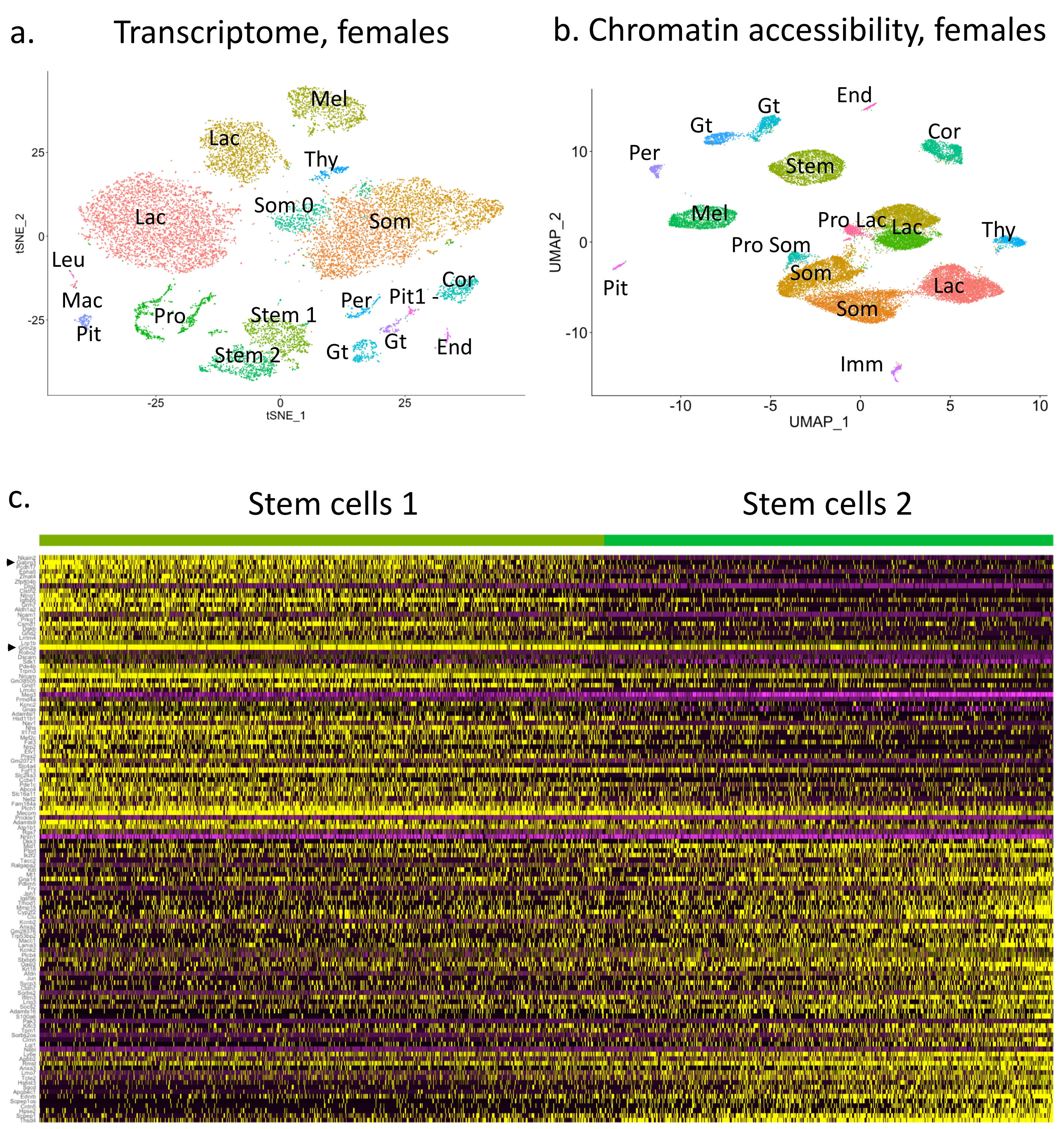

Supplementary Figure 2:

a. SnRNAseq merger of all female samples. Cell types are identified on the t-SNE plot. Multiple clusters of the same cell type were identified in lactotropes, gonadotropes, somatotropes, and stem cells.

b. SnATACseq merger of all female samples. Cell types are identified on the UMAP plot.

c. Heatmap showing the gradient of marker genes expression among stem cells in the snRNAseq merged snap-frozen females pituitary sample. A drop in stem cell 1 markers delimits the two subtypes. (See panel **a**).

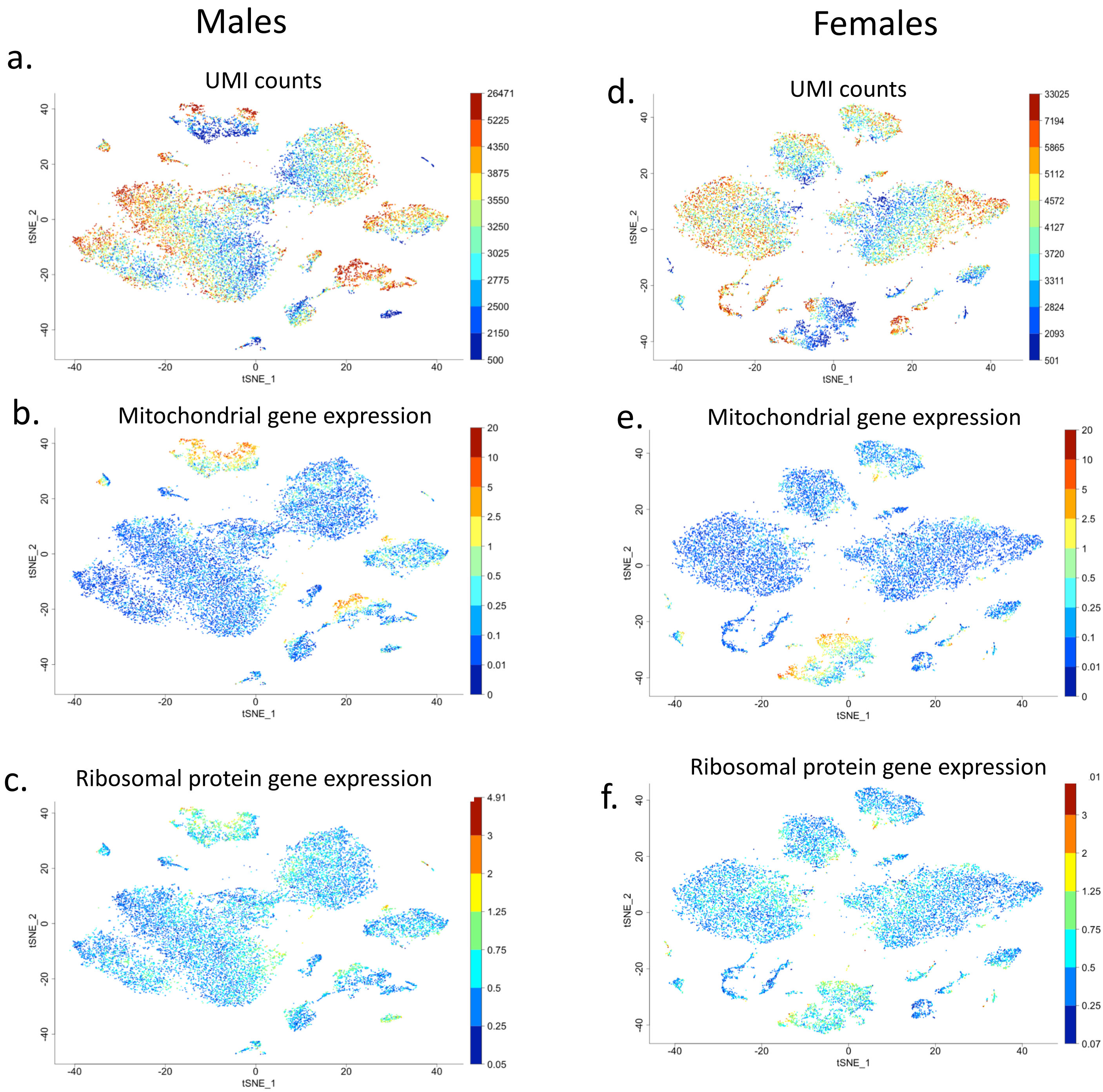

Supplementary Figure 3:
UMI counts (a,d), mitochondrial gene expression (b, e), and ribosomal protein gene expression (c, f) t-SNE for males (left) and females (right). Represented are percentages of total transcripts expressed.

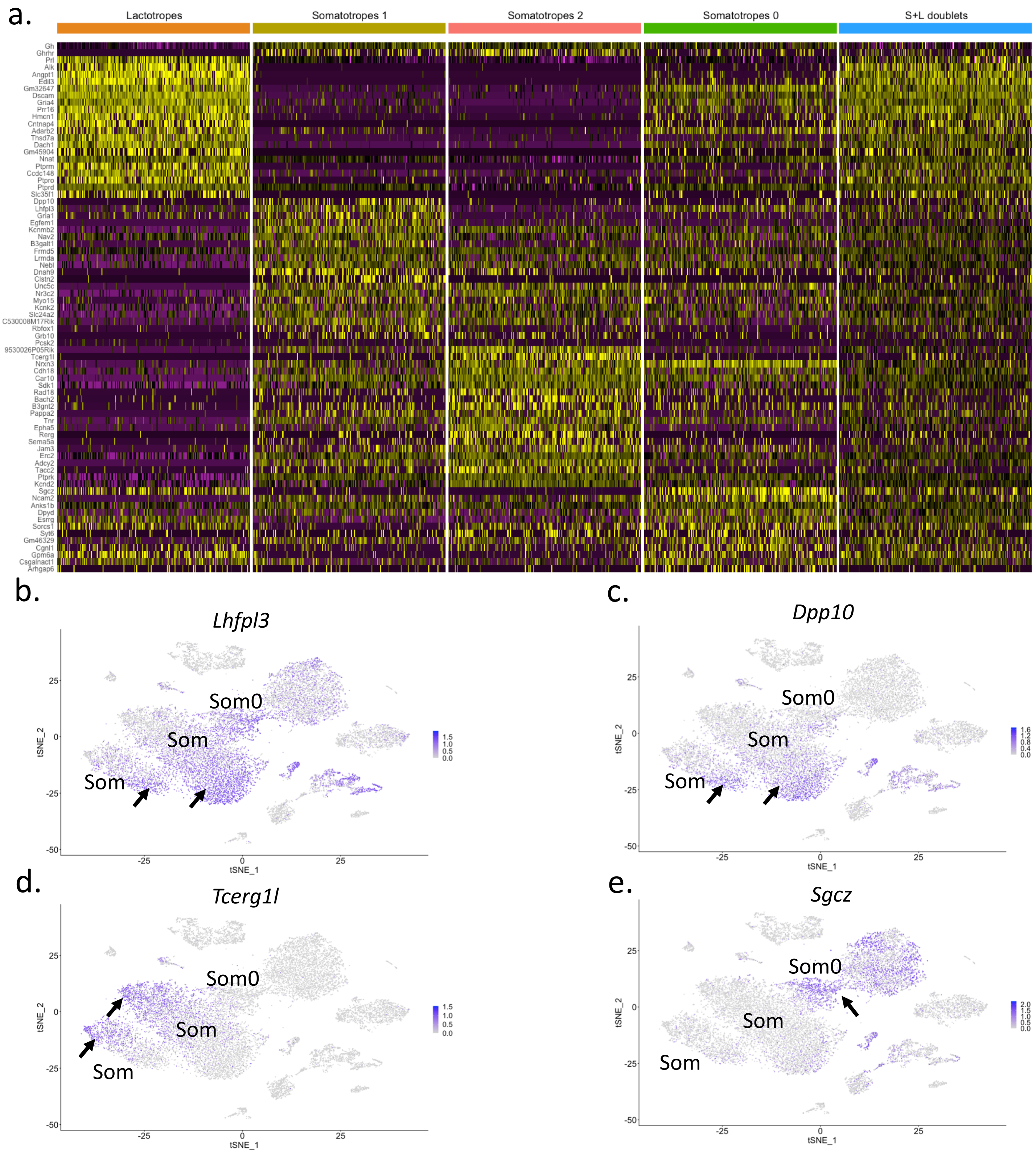

Supplementary Figure 4:
Analysis of the main somatotrope cluster and the link between somatotropes and lactotropes via somatotropes 0 (som 0) in the merged males sample. **a**. Heatmap of a subset of lactotropes, somatotrope sub-types, and somatotrope-lactotrope doublets showing expression of the top differentiating markers. **b-e**. t-SNE plots color-coded by the expression level of *Lhfpl3* and *Dpp10* (**b**, **c**) expressed at one pole (marked with an arrow) of the somatotrope cluster, *Tcerg1l* (**d**) expressed at the other pole, and *Sgcz* expressed in lacto-somatotropes, Som0 (e). Refer to **Supplementary Figures 32-34** for the t-SNE, UMI, and mitochondrial gene level analysis for this same sample.

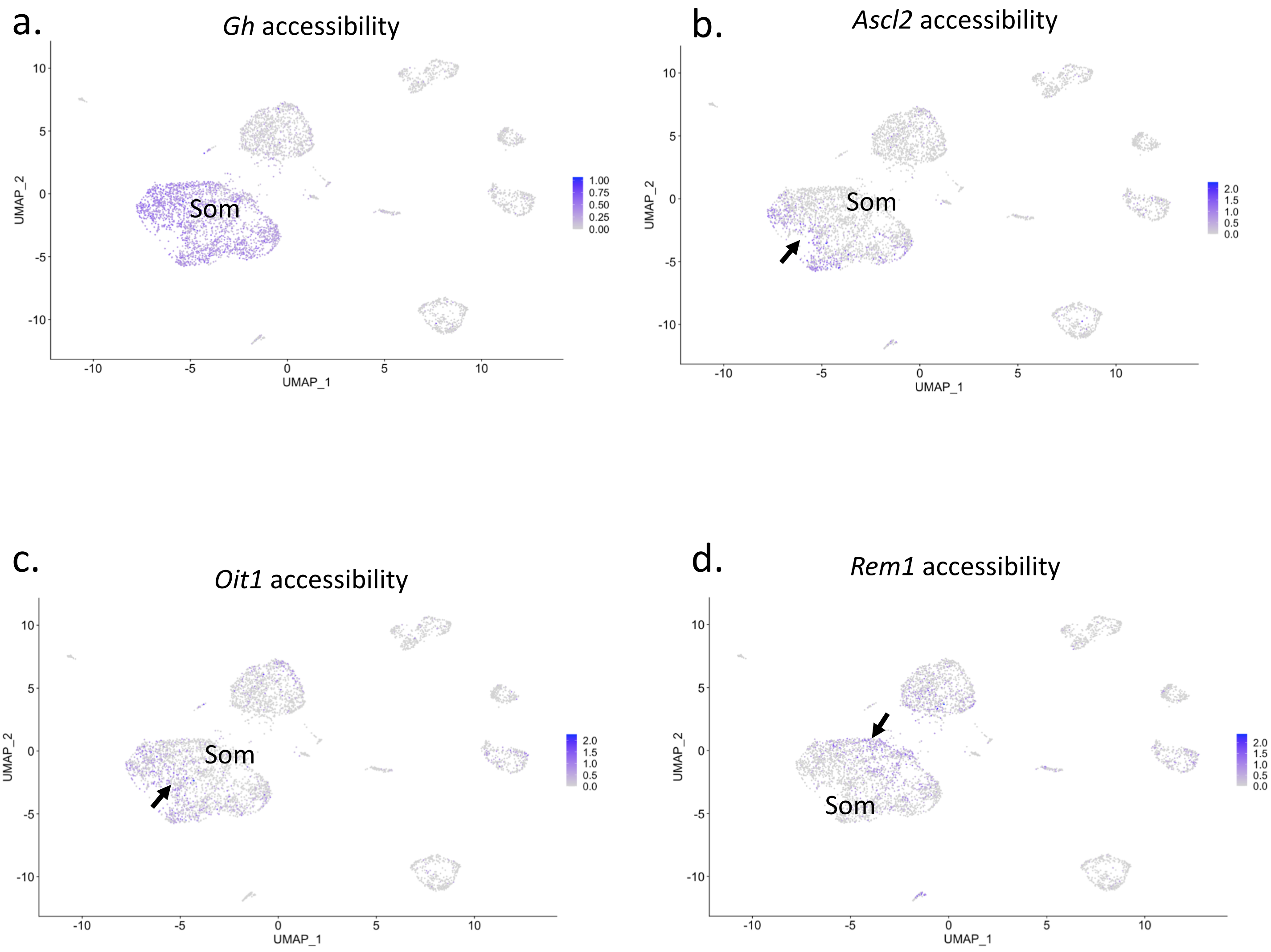

Supplementary Figure 5:
Analysis of the somatotrope cluster by snATACseq in the merged males sample illustrating **a**. homogeneous *Gh* gene accessibility throughout the cluster; **b,c**. accessibility of the *Ascl2* and *Oit1* promoters at one pole; **d**. accessibility of the *Rem1* promoter at the other pole.

a.

b.

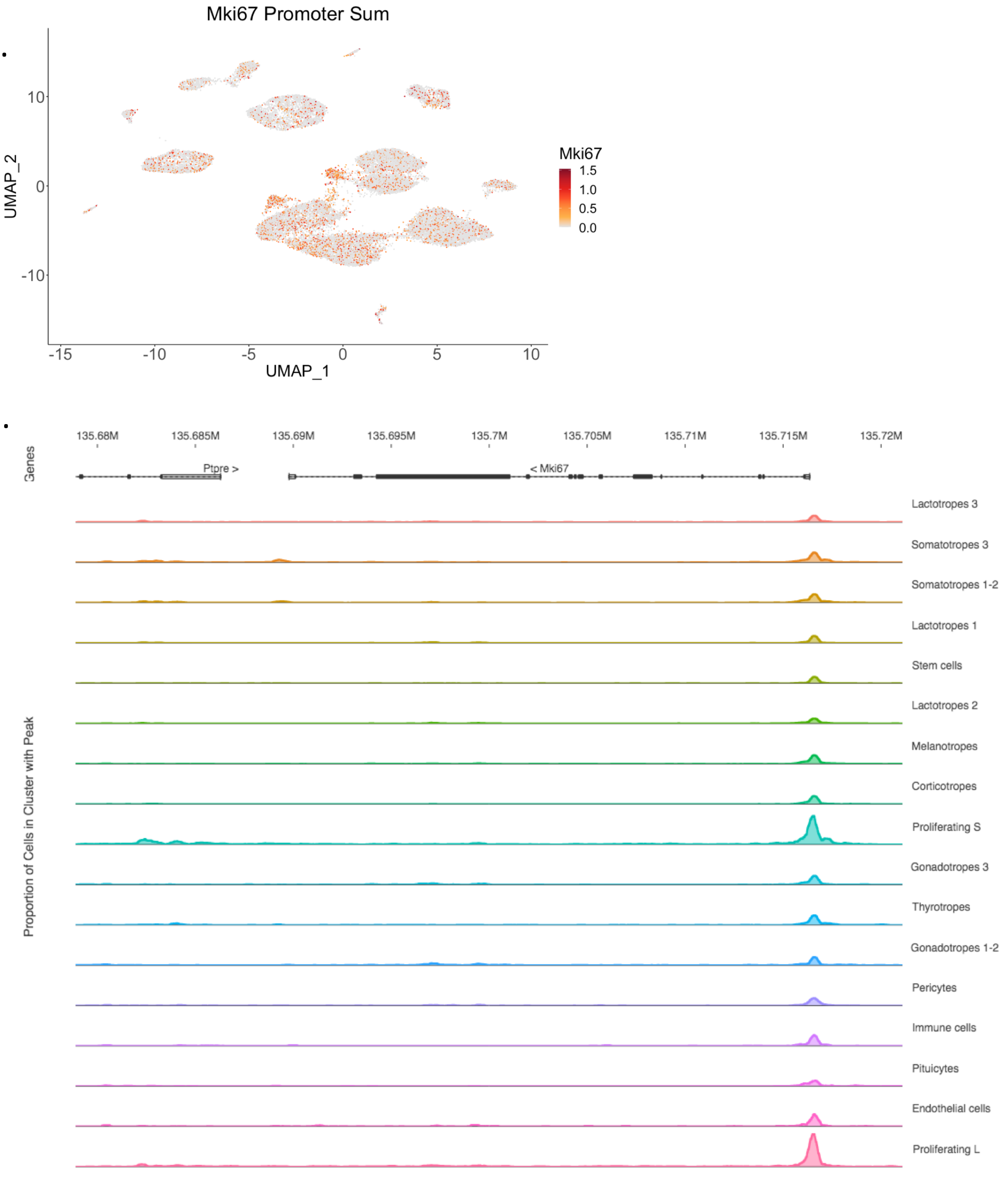

Supplementary Figure 6:
Analysis of the marker of proliferation *Mki67* promoter accessibility in the merged females snATACseq sample. **a.** Levels of promoter accessibility by cell shown on the UMAP projection. **b.** Chromatin accessibility tracks around the *Mki67* gene for each cluster. The females are identified by their animal number (female mouse 1 (1), female mouse 2 (2), female mouse 3 (3)).

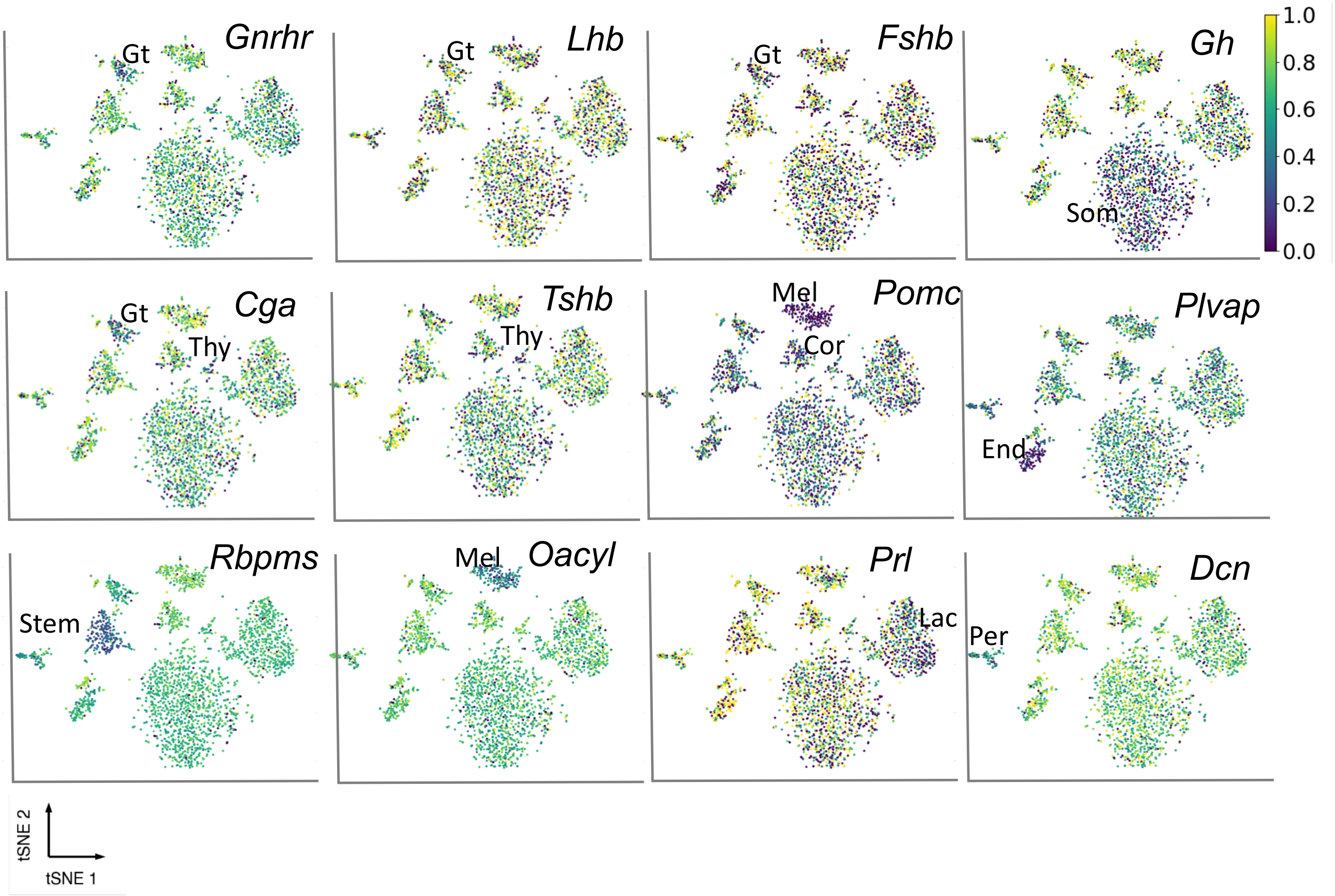

Supplementary Figure 7: Gene expression of key markers for the sn methylation assay.
Feature plots of key cell markers for cell cluster identification in the sn methylation dataset. The scale is presented on the top right corner.

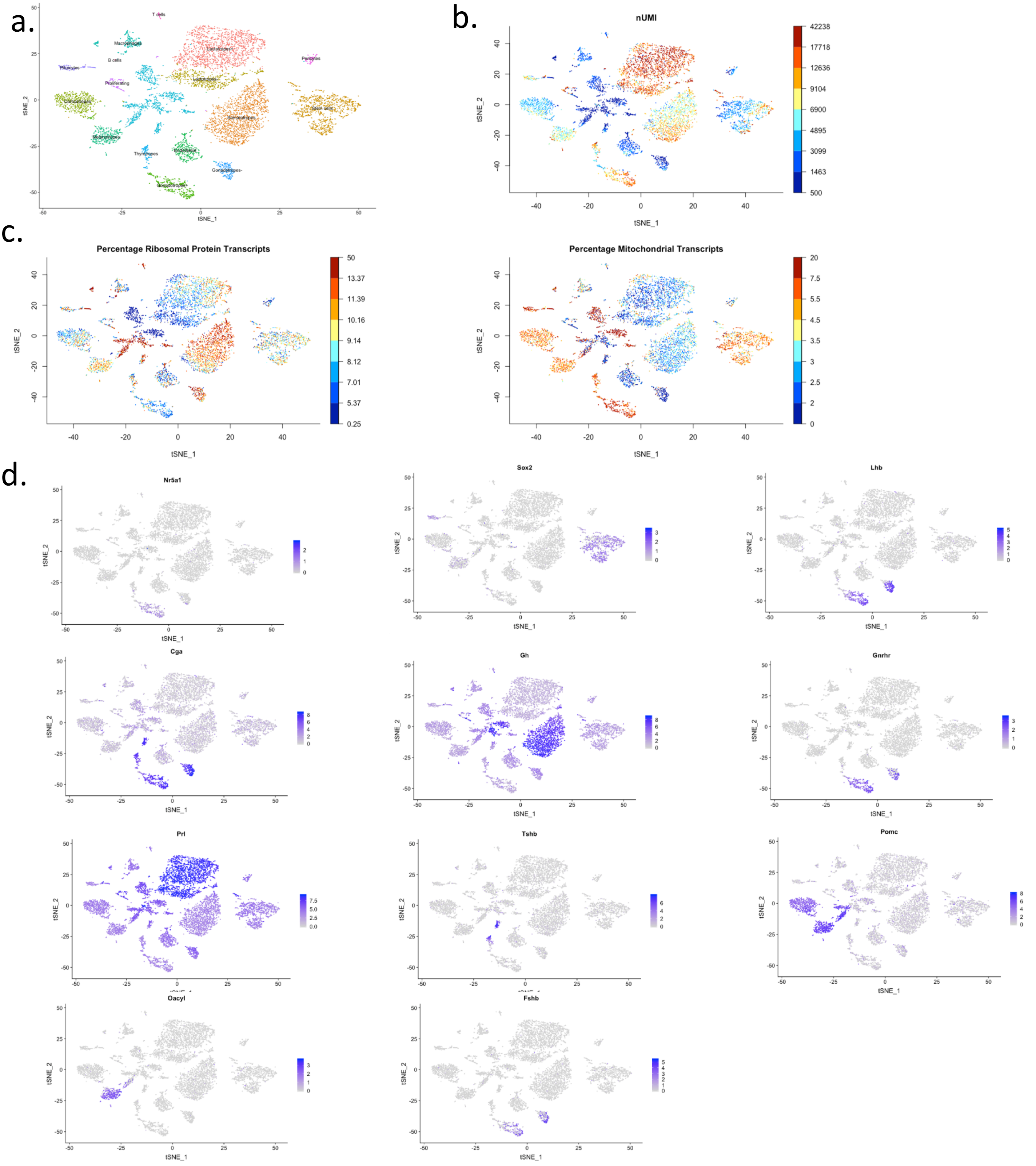

Supplementary Figure 8:
ScRNAseq analysis of a gently dissociated female pituitary. **a**. tSNE showing the different clusters obtained. **b**. Analysis of UMI content. **c**. Analysis of ribosomal content (Left panel), and mitochondrial content (Right panel). **d**. Feature plots showing the main pituitary markers for cell identification. A similar analysis was repeated in another dissociated female, whereas in dissociated male samples a different protocol was used (refer to **Supplementary Figure 1**). See **Supplementary Figure 9** for the same sample analyzed by snRNAseq, and **Supplementary Figure 14** for the same sample analyzed by snATACseq.

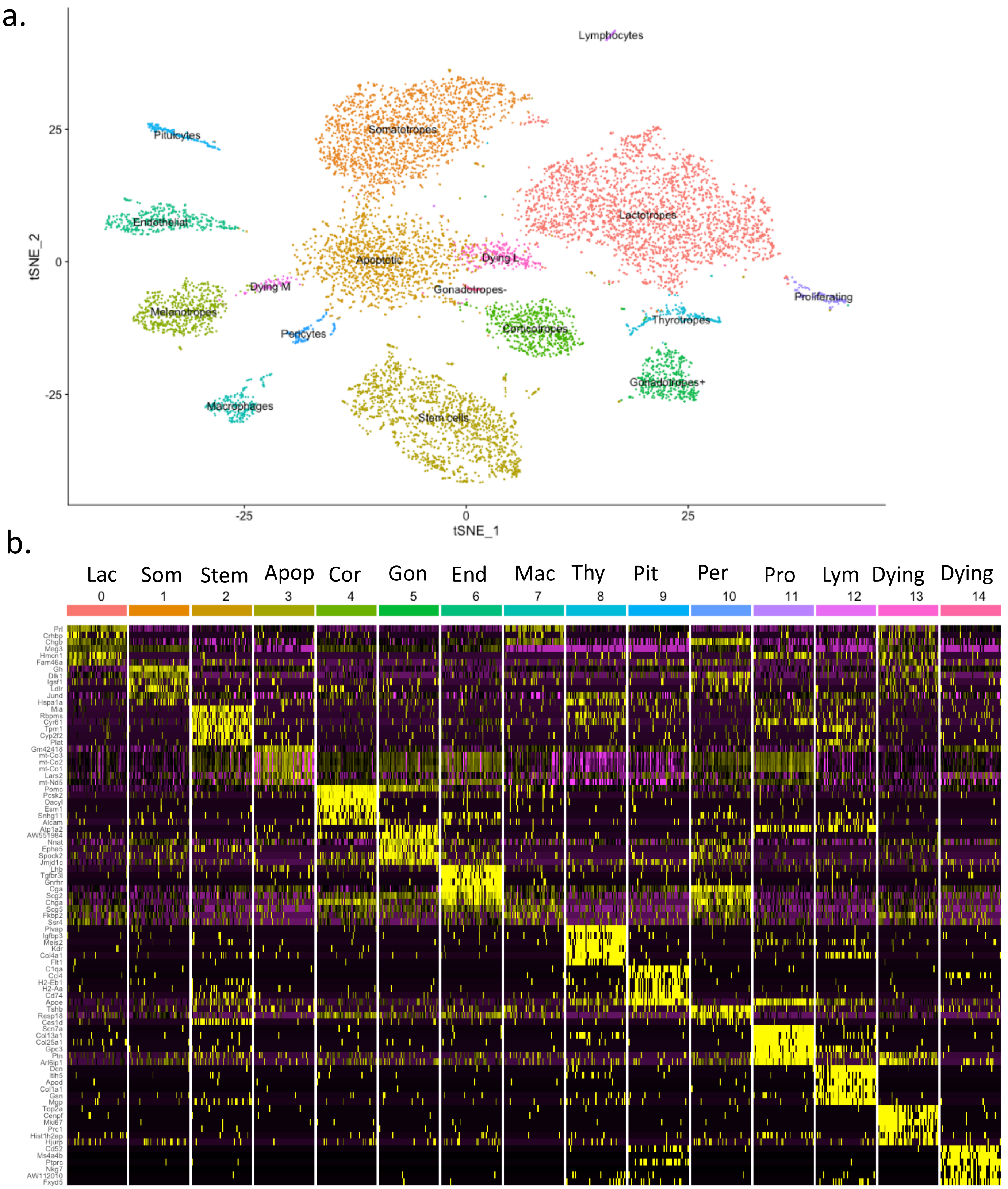

Supplementary Figure 9:
snRNAseq analysis of a gently dissociated female pituitary (refer to **Supplementary Figure 8** for the same sample analyzed by scRNAseq). **a**. t-SNE showing the different clusters that were obtained. **b**. Heatmap of showing cell identity based on gene expression in the sample. Note that contrary in contrast to scRNAseq (**Supplementary Figure 8**) we do not see the low UMIs cell clusters with snRNAseq. Also refer to **Supplementary Figure 10** for samples analyzed by snRNAseq from snap-frozen pituitary.

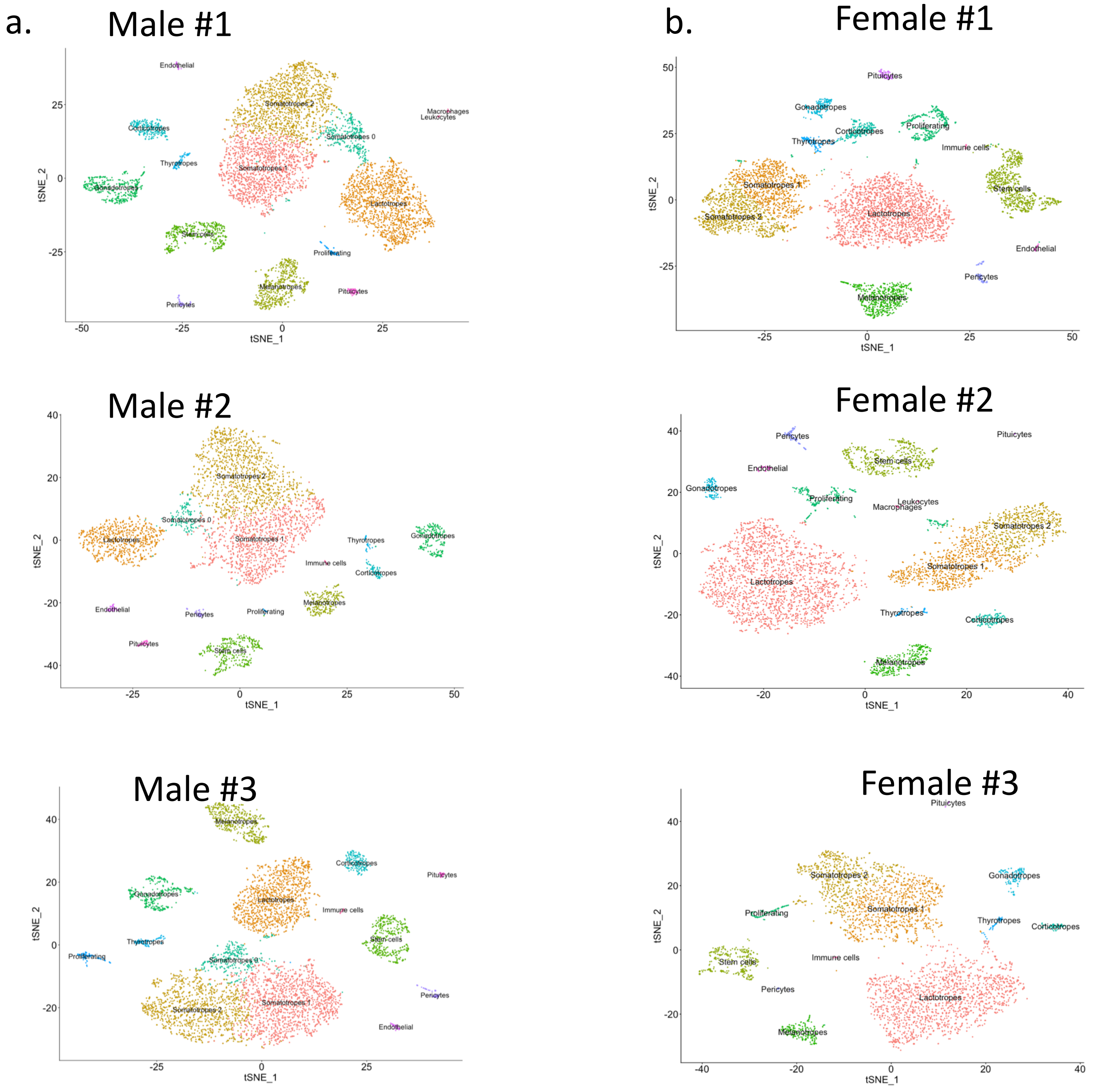

Supplementary Figure 10:
Results obtained for the snap-frozen pituitaries that were processed for snRNAseq. t-SNE of all individual animals processed for snRNAseq from individually snap-frozen pituitaries (3 males, panel a; and 3 females, panel b). The main somatotrope cluster is presented with the two subtypes Som1 and Som2 representing the two poles. Refer to **Supplementary Figures 32-34** for the t-SNE, UMI, and mitochondrial gene level analysis for all samples.

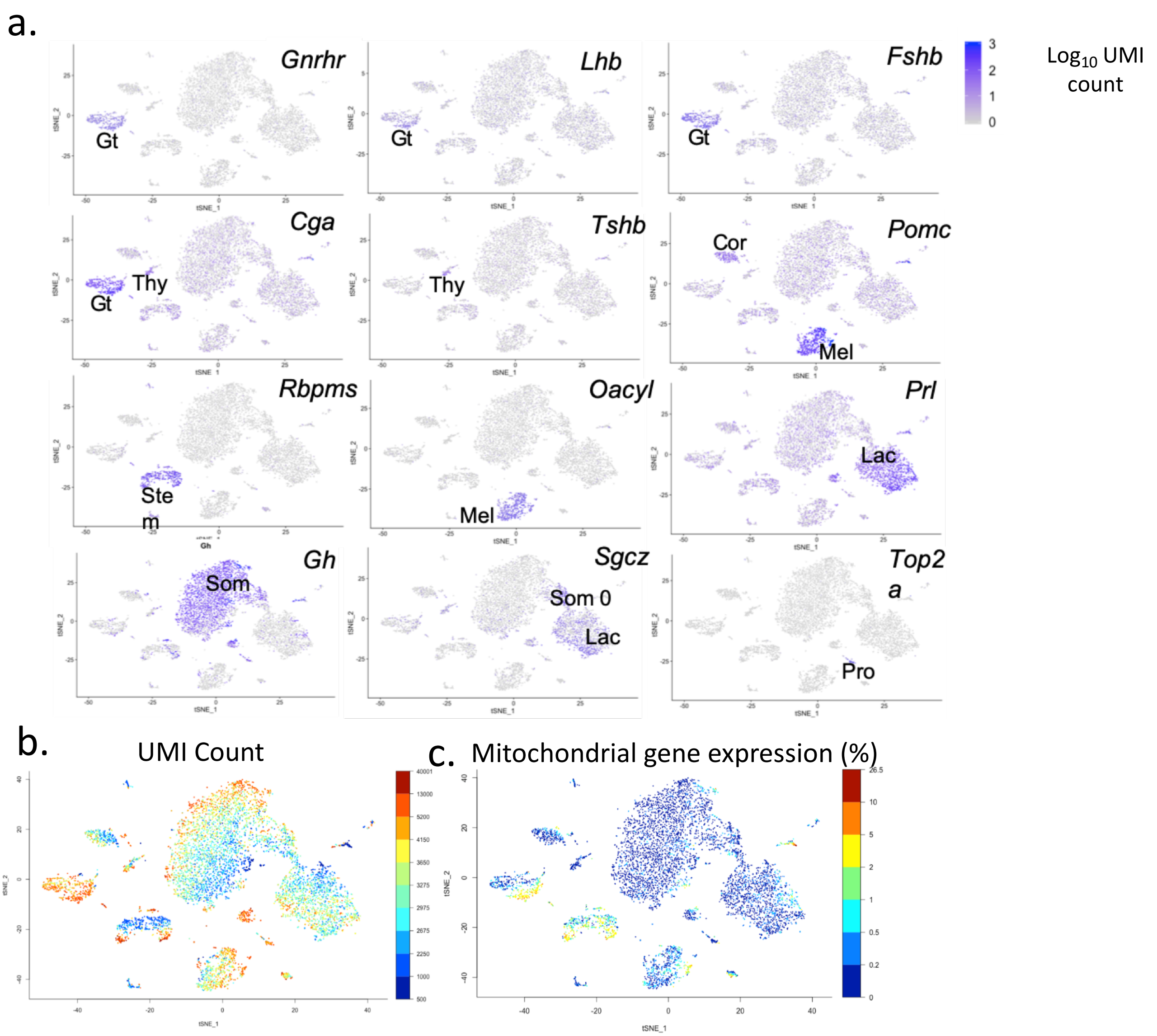

Supplementary Figure 11:
Identification of mouse pituitary cell types by sn transcriptomic analysis
a. Individual feature plots for transcripts used to identify major cell types in one male pituitary. **b**. UMI count graph showing the number of individual transcripts in one male pituitary. **c**. Graph showing mitochondrial gene expression in one male pituitary. **a-c** show the same male pituitary (Mouse 1).

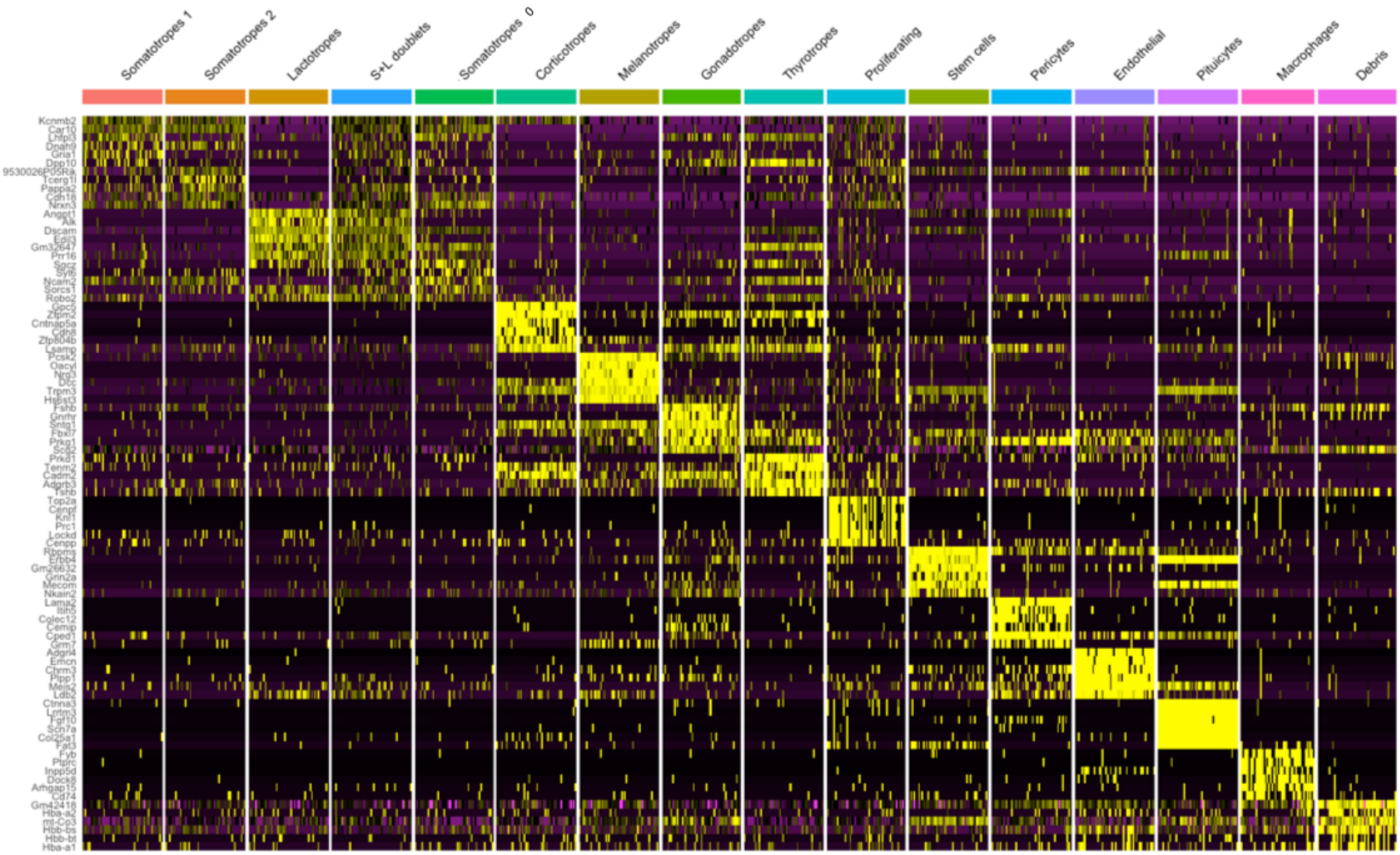

Supplementary Figure 12:
Heatmap showing cell identity based on gene expression in one male snap-frozen male pituitary from snRNAseq. Refer to **Supplementary Figure 9** for a similar heatmap from nuclei isolated from dissociated cryopreserved cells.

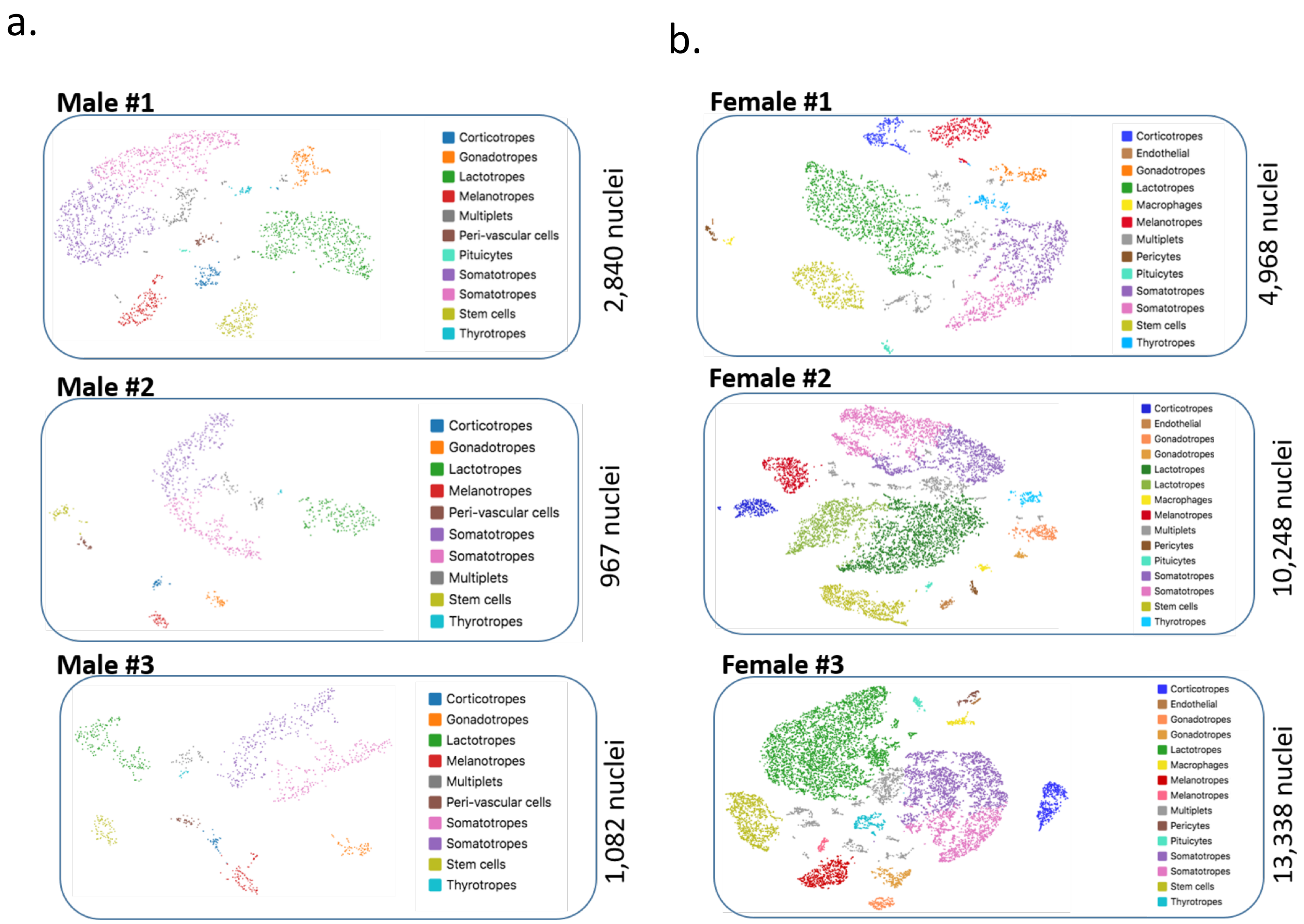

Supplementary Figure 13:
Results obtained for snap-frozen pituitaries that were processed for snATACseq. **a**. tSNE of all individual animals processed for snATACseq from individually snap-frozen pituitaries (3 males, panel a; and 3 females, panel b). These are the same samples that were processed for snRNAseq (**Supplementary Figure 10**).

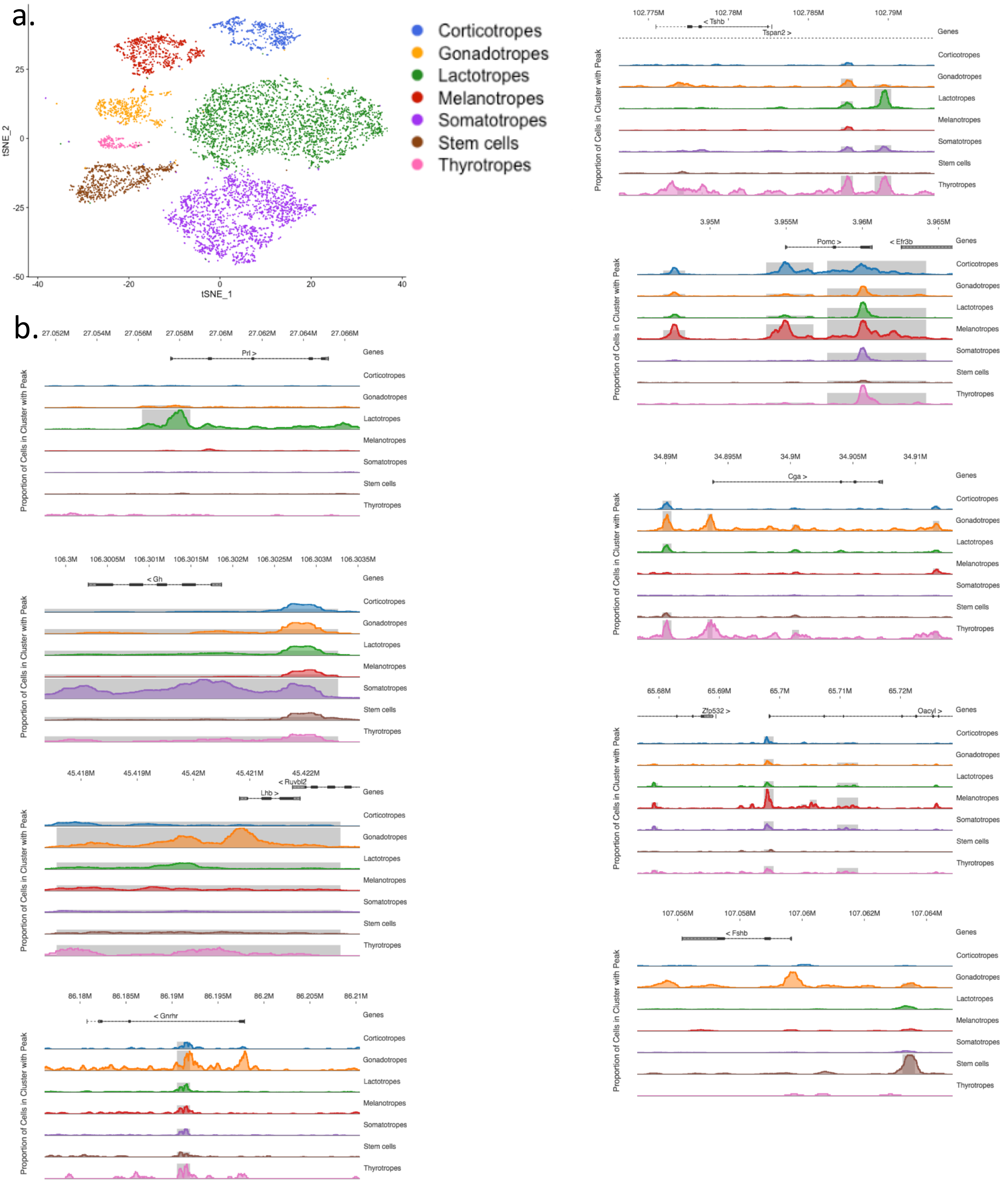

Supplementary Figure 14:

SnATACseq analysis of a gently dissociated female pituitary (refer to **Supplementary Figure 8** for the same sample analyzed by scRNAseq, and **Supplementary Figure 9** for the same sample analyzed by snRNAseq). **a**. tSNE identifying the different clusters that were obtained. **b**. Chromatin accessibility tracks around canonical markers of pituitary cell-types.

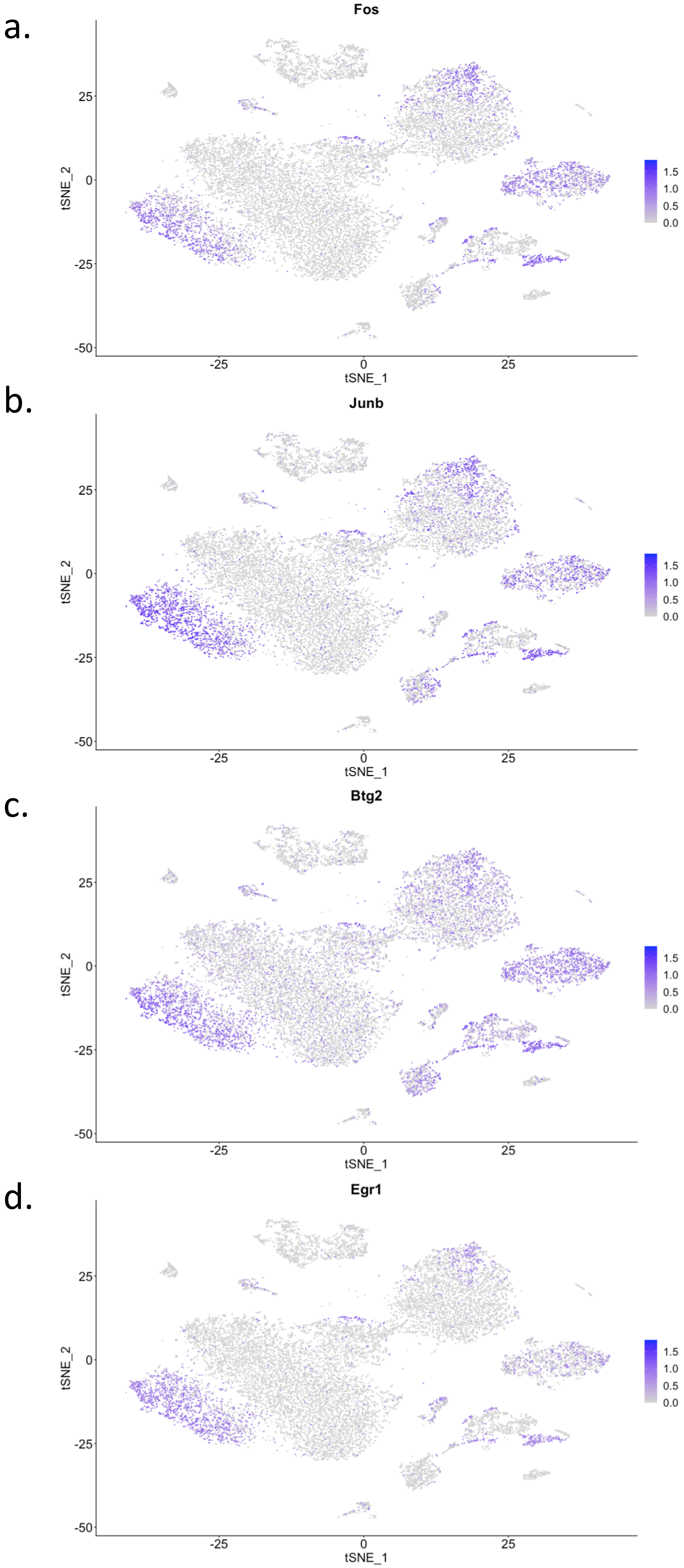

Supplementary Figure 15: Feature plots in merged males sample showing *IEGs* expression. *Fos* (a), *Junb* (b), *Btg2* (c), and *Egr1* (d).

a.

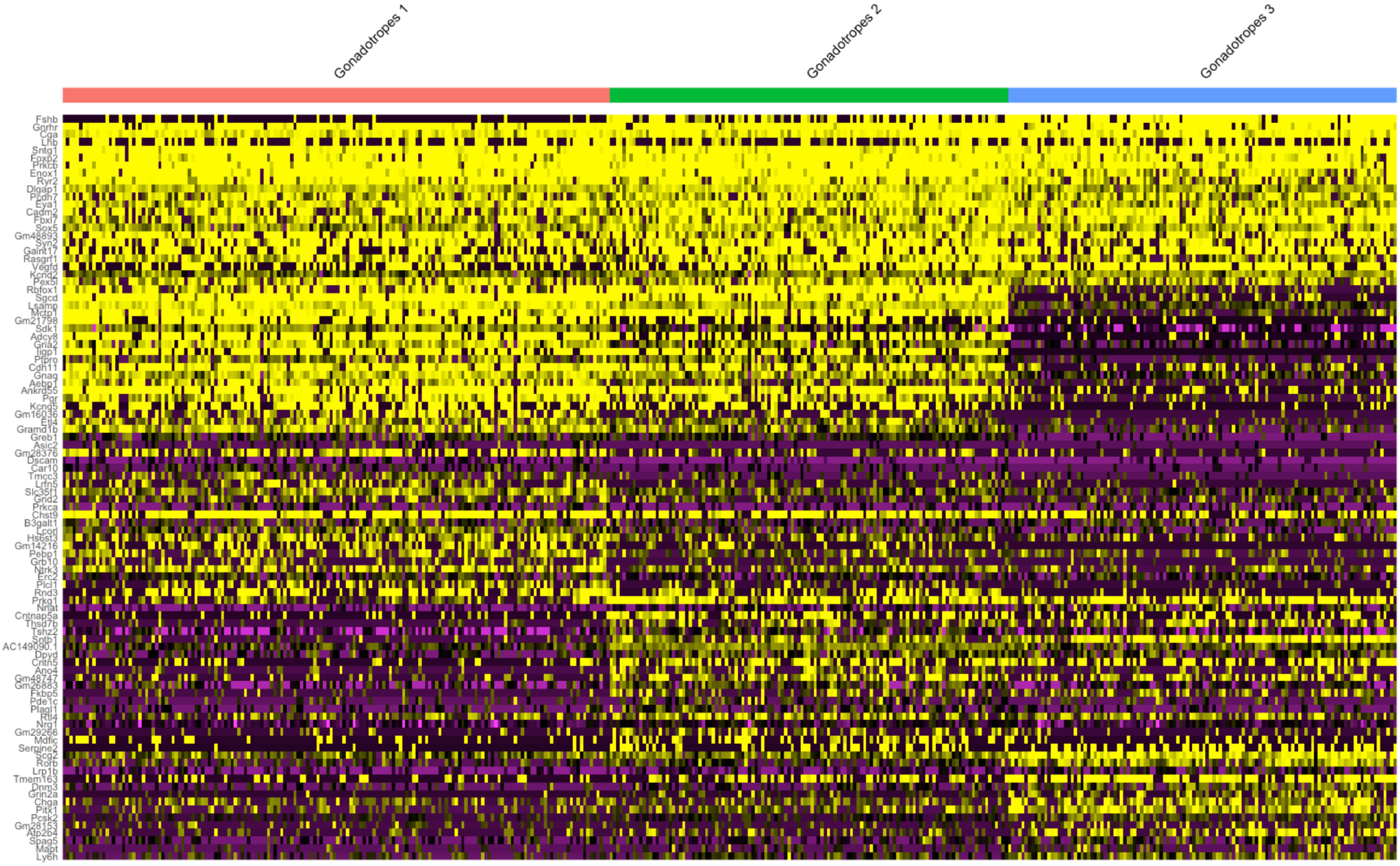

b.

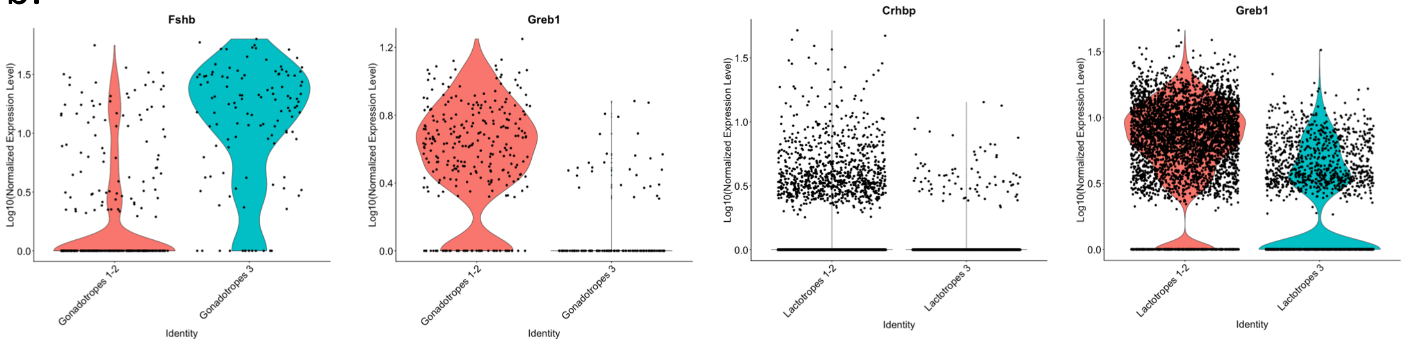

Supplementary Figure 16:

Markers differentiating individual animals in the merged analysis of female samples. **a**. Heatmap showing common and differentiating markers of gonadotropes. **b**. Examples of differential gene expression among gonadotropes and lactotropes. The individual females are identified by their number (1-3).

LV1rna, sex differences

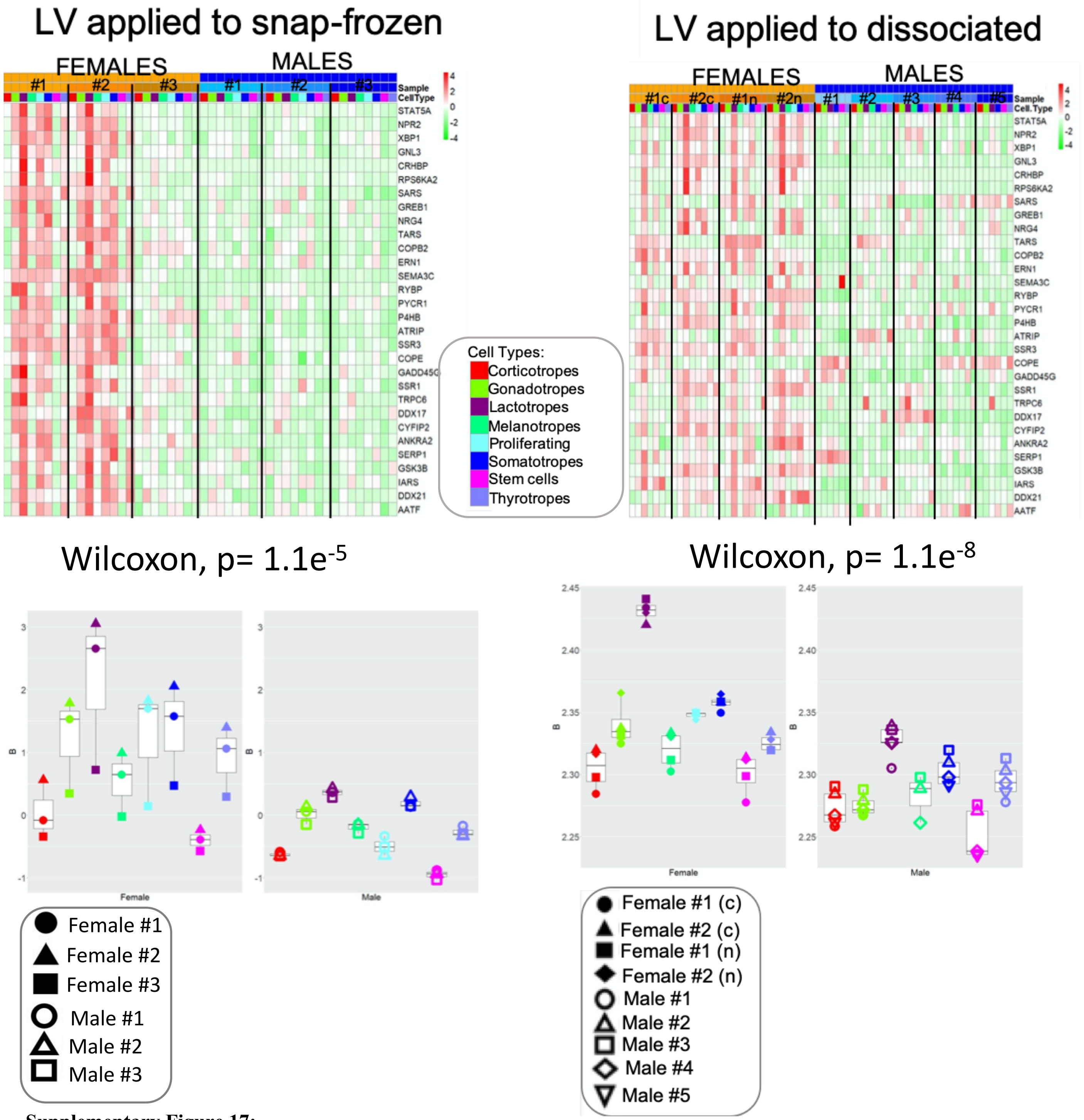

Supplementary Figure 17:

PLIER analysis showing comparison of the sex differentiating LV (LV1rna) in snap-frozen pituitaries (Left panels), vs. in dissociated and cryopreserved pituitaries (Right panels, also presented in **Supplementary Figure 21**). Top panels: heatmaps showing the top 30 genes of the sex differentiating LV1rna. Bottom panels: boxplots showing the statistical analysis. LV1 RNAseq showed increased expression in females compared to males in the snap-frozen pituitaries (Wilcoxon $p=1.1e^{-5}$; refer to **Supplementary Figure 7b**), as well as in the dissociated pituitaries (Wilcoxon $p=1.1e^{-8}$). Dissociated female pituitary sample were analyzed by scRNAseq (c) and snRNAseq (n). Dissociated male pituitary samples were analyzed in scRNAseq only. A definition of B score is provided in the Methods. For the box plots, n=3 independent animals were used for the first two panels, n=4 for the third panel and n=5 for the fourth panel. ggboxplot generates a boxplot with the center equal to the 50th percentile, the bounds of the box are the 25th and 75th percentile and the bounds of the whiskers are the smallest/largest values 1.5 times the interquartile range below the 25th percentile or above the 75th percentile, respectively. Statistical analysis was done with the Wilcoxon ranked-sum test.

Fshb

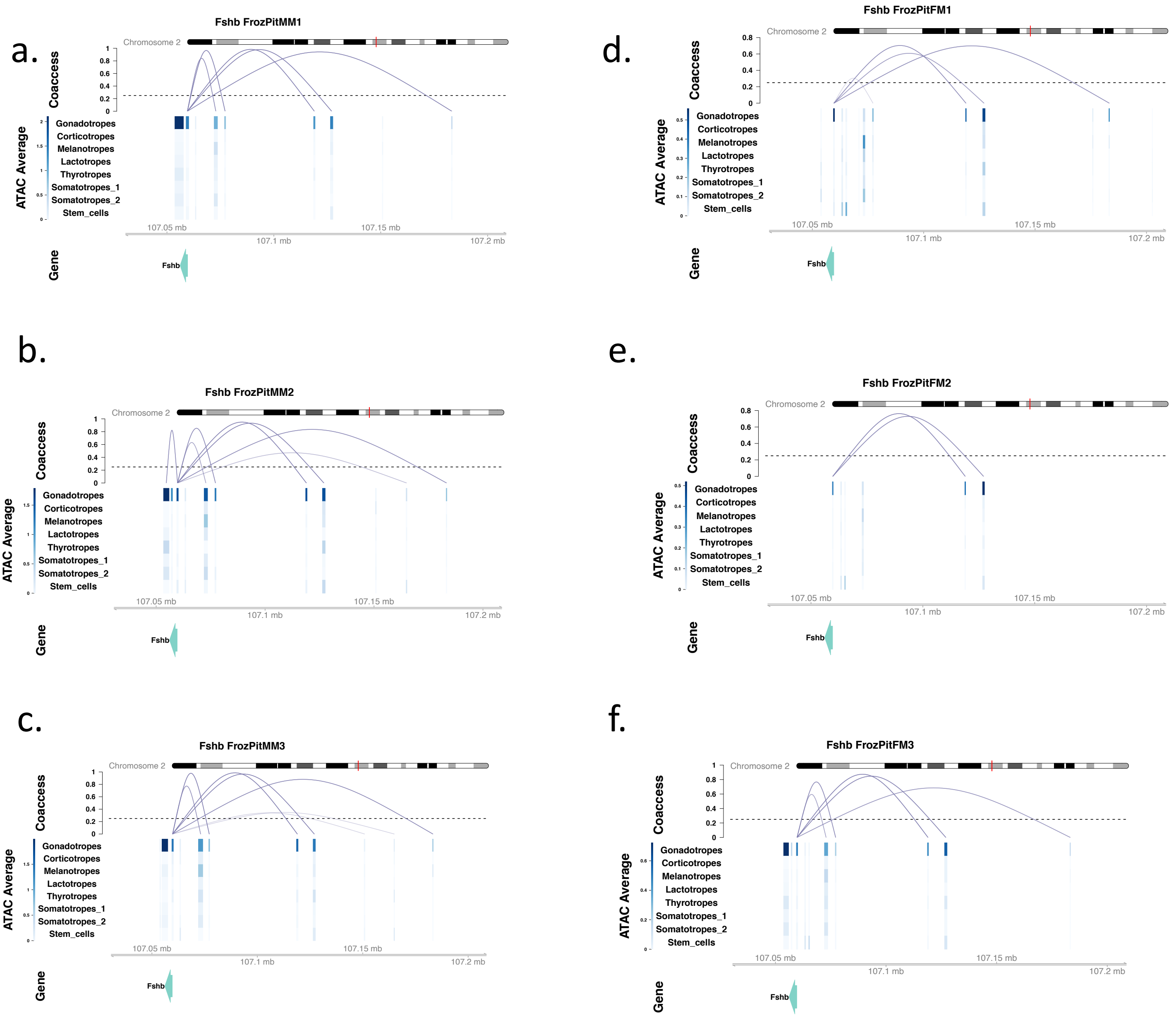

Supplementary Figure 18:
Co-accessibility analysis for the *Fshb* promoter in all 6 individual snap-frozen pituitaries (3 males (**a-c**) and 3 females (**d-f**)). Refer to **Fig. 6a**.

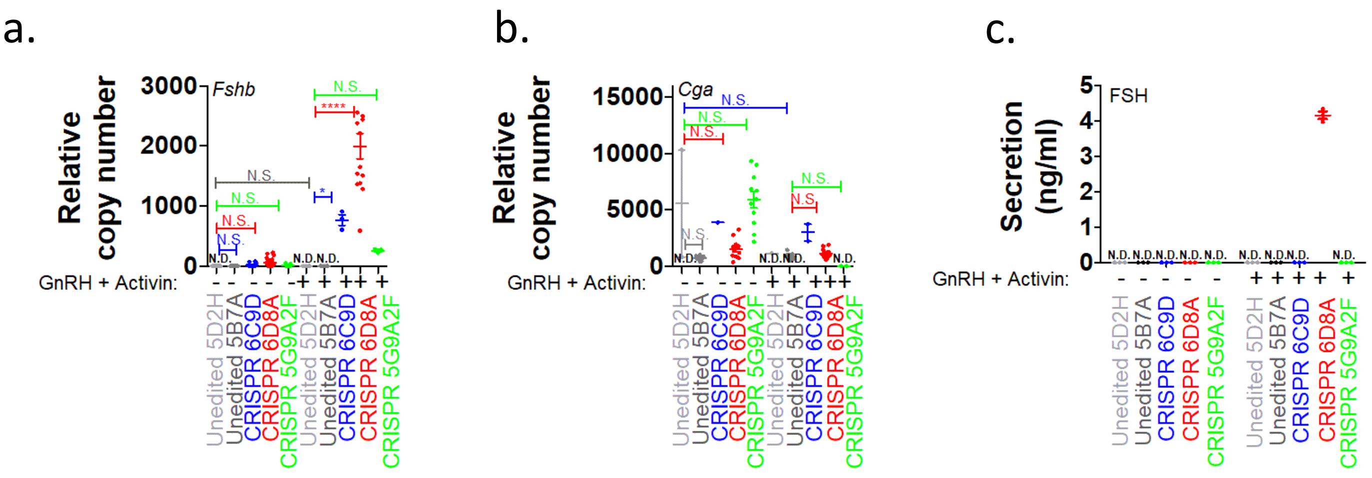

Clone Name	Edited sequence ATATTTATTTCA*G*GG
**5D2H**	Wild type unedited
**5B7A**	Wild type unedited
**6D8A**	Del TTATTT
**6C9D**	Del TTT
**5G9A2F**	Del T/Insert TATT

Supplementary Figure 19:
Shown are *Fshb* (a, Left panel) and *Cga* (b, Center panel) gene expression, and FSH secretion levels (c, Right panel) in unedited gonadotrope cell line clones either at baseline (-), or following GnRH(grey, clone 5D2H and 5B7A) and in three CRISPR deletion mutant lines (blue clone 6C9D, red clone 6D8A, green clone 5G9A2F), and activin A treatment (+). Each data point is shown. Data are from 6 independent experiments. Error bars represent s.e.m. Significance was determined by 2-way ANOVA with Bonferroni corrections. N.S: not significant, $p>0.05$; *: $p<0.1$; **: $p<0.01$; ****: $p<0.0001$. N.D.: Not detected. Presented below is a table identifying all the clones and their edited sequence.

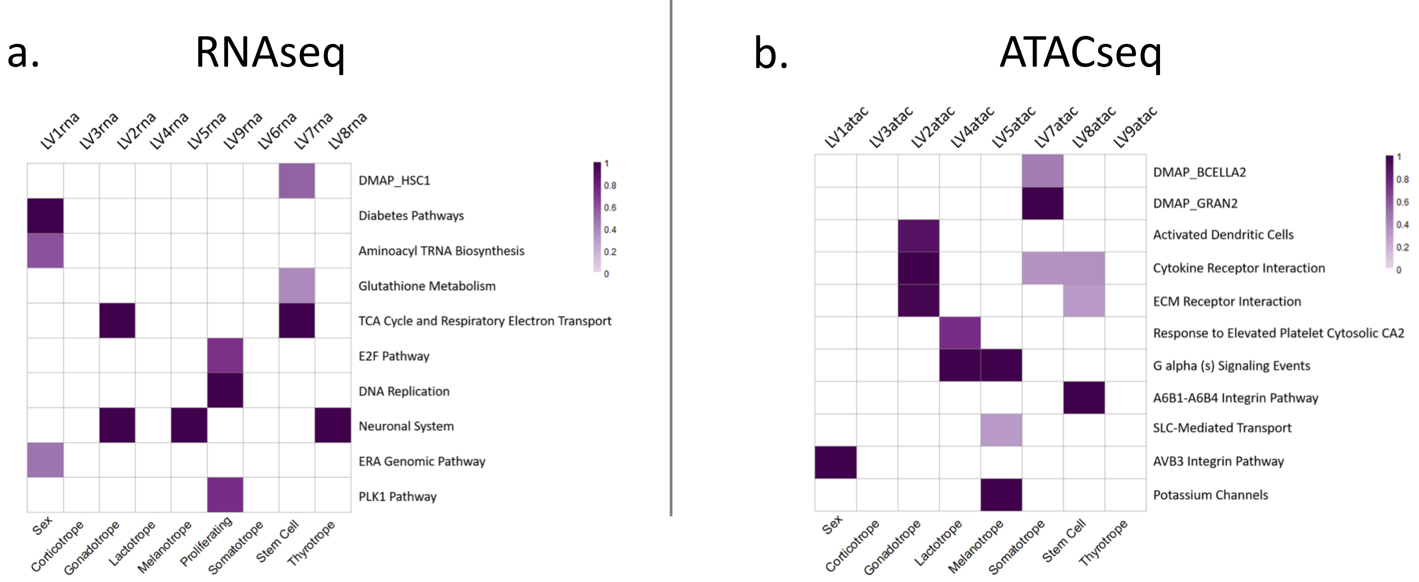

Supplementary Figure 20:
a,**b**. PLIER analysis of the snRNAseq (**a**.) and snATACseq data (**b**.) from the six individual snap-frozen pituitaries. The panel shows the pathways associated with each sex or cell type-specific latent variable (LV) obtained from the given PLIER analysis. Refer to **Fig. 7**.

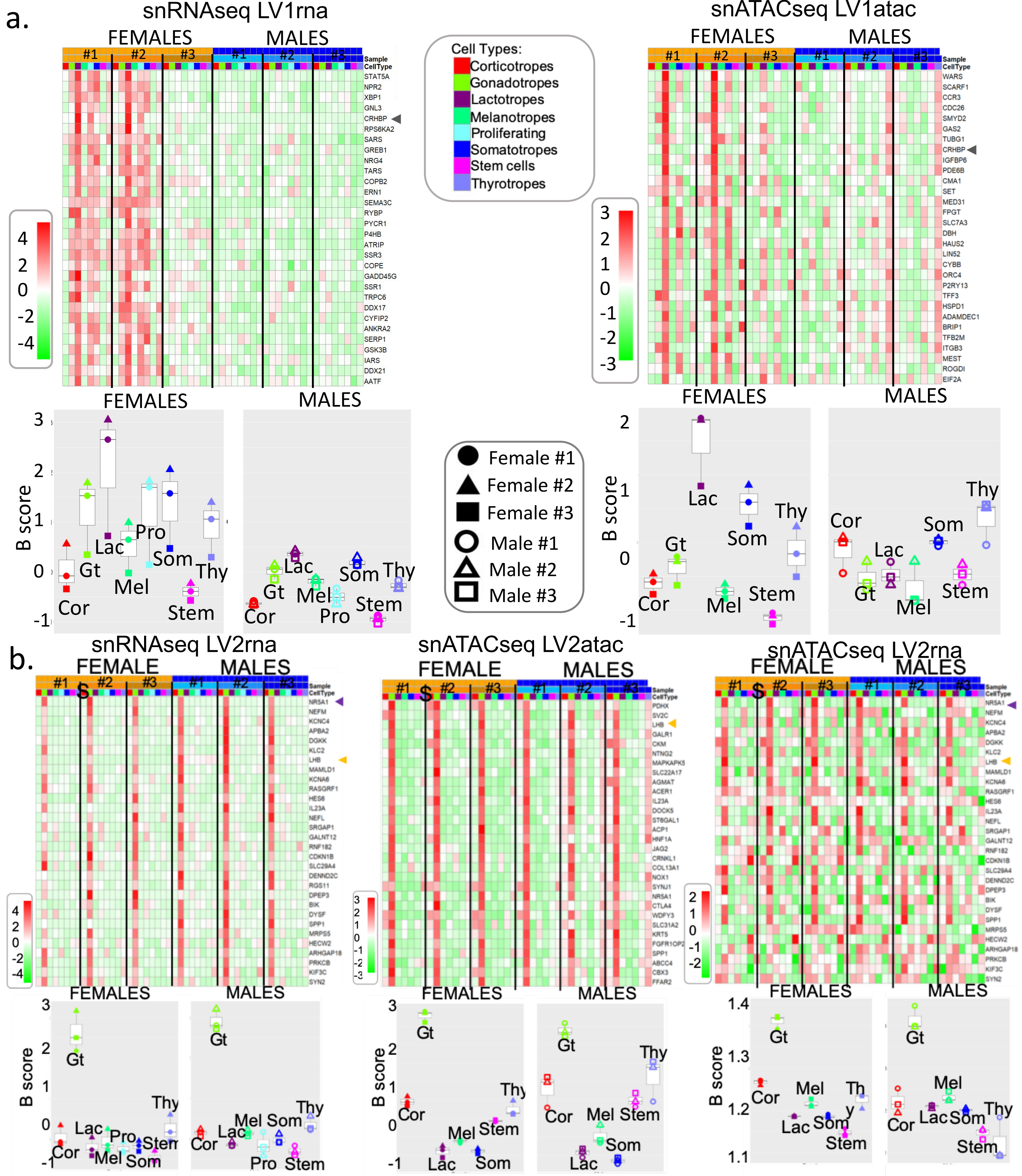

Supplementary Figure 21: Inter-animal differences in gene expression and chromatin accessibility

a. Identification of LVs showing sex and inter-animal differences using PLIER. LV1rna (left panel) and LV1atac (right panel) were inferred from the snRNAseq and snATACseq data, respectively. Depicted are a heatmap (top) and expression levels (bottom) of each LV across samples and cell types. Differences between sexes were significant for LV1rna (p= 1.1e-5, Wilcoxon, all cell types), and for LV1atac (p= 0.02 for lactotropes only). A top driver gene is indicated (grey arrow). **b**. Heatmap and expression levels of an LV that is elevated in gonadotropes. The LV was identified from the snRNAseq data (left panel, LV2rna), applied to snATAC data (right panel, LV2rna), and a similar LV was identified from the snATAC data (center panel, LV2atac). A scale bar is provided on the left-hand side of each heatmap, with red being the highest gene expression or most accessible chromatin. Selected genes that are characteristic of gonadotropes are indicated with arrows. A definition of B score is provided in the Methods. For the box plots, n=3 independent animals were used for all panels. ggboxplot generates a boxplot with the centre equal to the 50th percentile, the bounds of the box are the 25th and 75th percentile and the bounds of the whiskers are the smallest/largest values 1.5 times the interquartile range below the 25th percentile or above the 75th percentile, respectively. Statistical analysis was done with the two-sided Wilcoxon ranked sum test for the boxplots. -

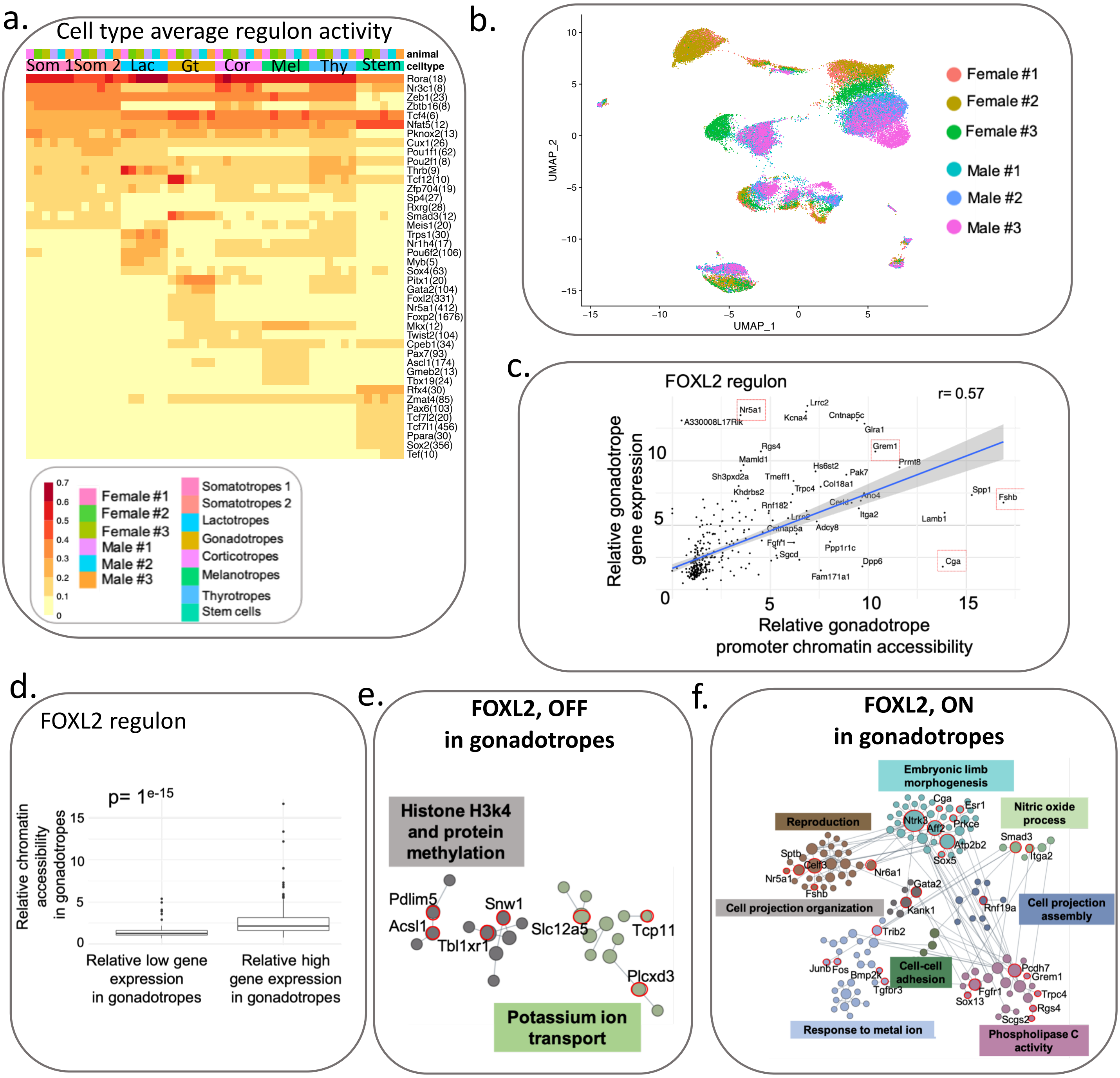

Supplementary Figure 22: Gonadotrope-specific regulon activity

a. Heatmap of the SCENIC cell type average regulon activity scores per animal. The color scale for the heatmap, samples and cell type legend is presented below the heatmap. **b**. UMAP of the individual animal samples overlaid on the cell clusters obtained with the SCENIC analysis shows animal variations (**Fig.8c**). **c**. Scatter plot and correlation of the 331 genes composing the FOXL2 regulon based on their relative gene expression and relative TSS chromatin accessibility in male gonadotropes. Selected genes are boxed in red. **d**. Boxplots of the relative chromatin accessibility within gene bodies for the FOXL2 regulon separated into relatively low expression vs. relatively high expression in male gonadotropes. Two-sided Wilcoxon rank-sum tests (W = 3388). Left (low): min 0.72, max 5.39, lower whisker 0.72, lower box 1.10, median 1.30, upper box 1.65, upper whisker 2.21 Right (high): min 0.81, max 16.63, lower whisker 0.81, lower box 1.63, median 2.17, upper box 3.16, upper whisker 5.32 **e**. Annotated gene modules based on FOXL2 regulon genes that are not expressed (off) in male gonadotropes. **f**. Annotated gene modules based on FOXL2 regulon genes expressed (on) in male gonadotropes. Panels **c-f** are based on data from 3 individual male samples merged as a consensus male animal. See **Supplementary Figure 25** for corresponding analysis of female samples and **Supplementary Figure 27** for another gonadotrope-specific regulon NR5A1. Refer to the Method section for the definition of ‘relatively high’ and ‘relatively low’.

FOXP2 regulon, male stem cells

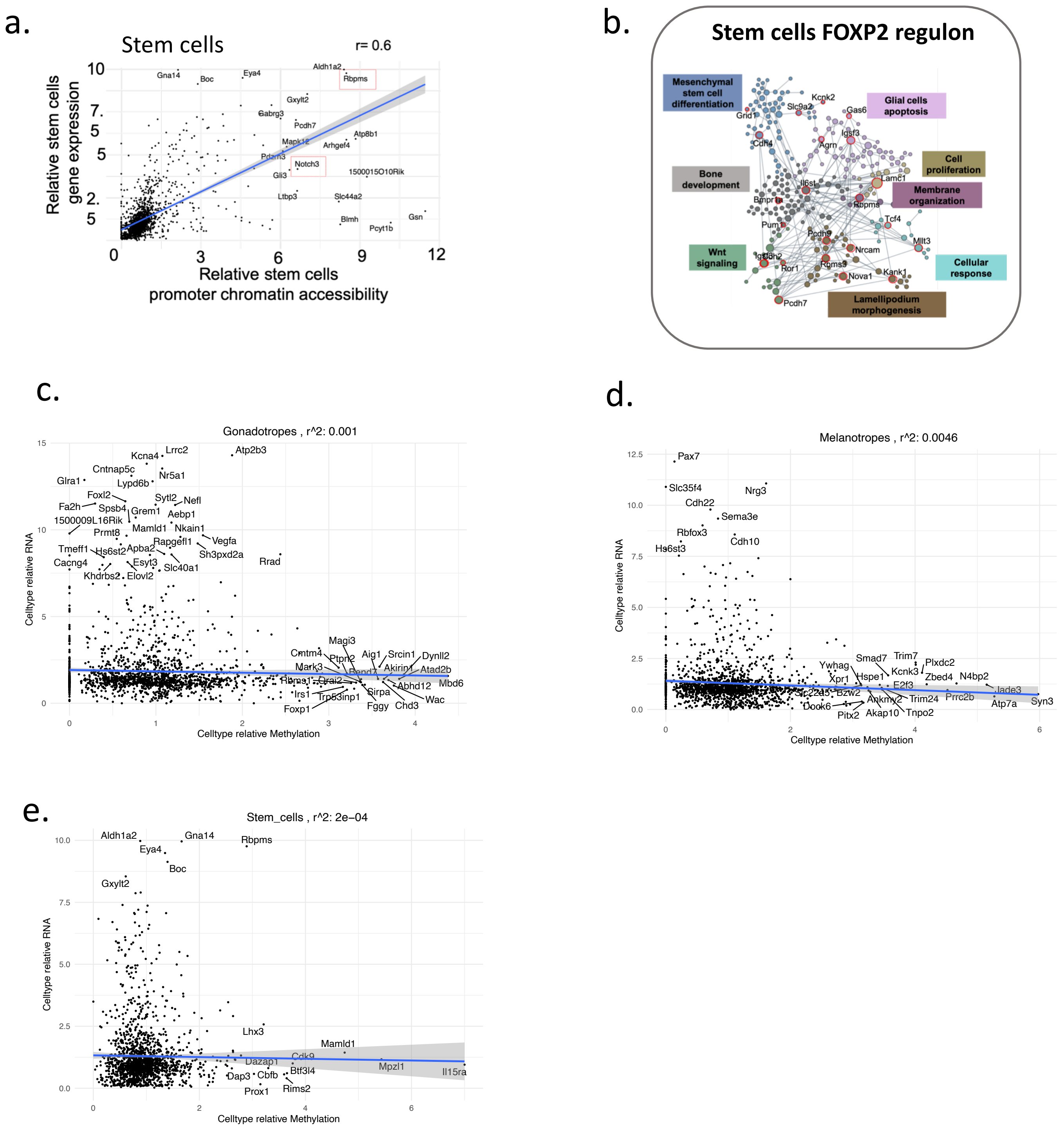

Supplementary Figure 23:

a. Scatter plot and correlation of the genes composing the FOXP2 regulon based on their relative gene expression and relative TSS chromatin accessibility in male stem cells (consensus male from 3 individual male samples). Boxed in red are genes that are related to stem cell regulation. **b**. Gene network for the FOXP2 regulon targets that are ON in male stem (consensus male from 3 individual male samples). **c-e**. Scatter plot and correlation of the genes composing the FOXP2 regulon based on their relative gene expression and relative methylation in male gonadotropes (c), melanotropes (d), and stem cells (e; consensus male from 3 individual male samples).

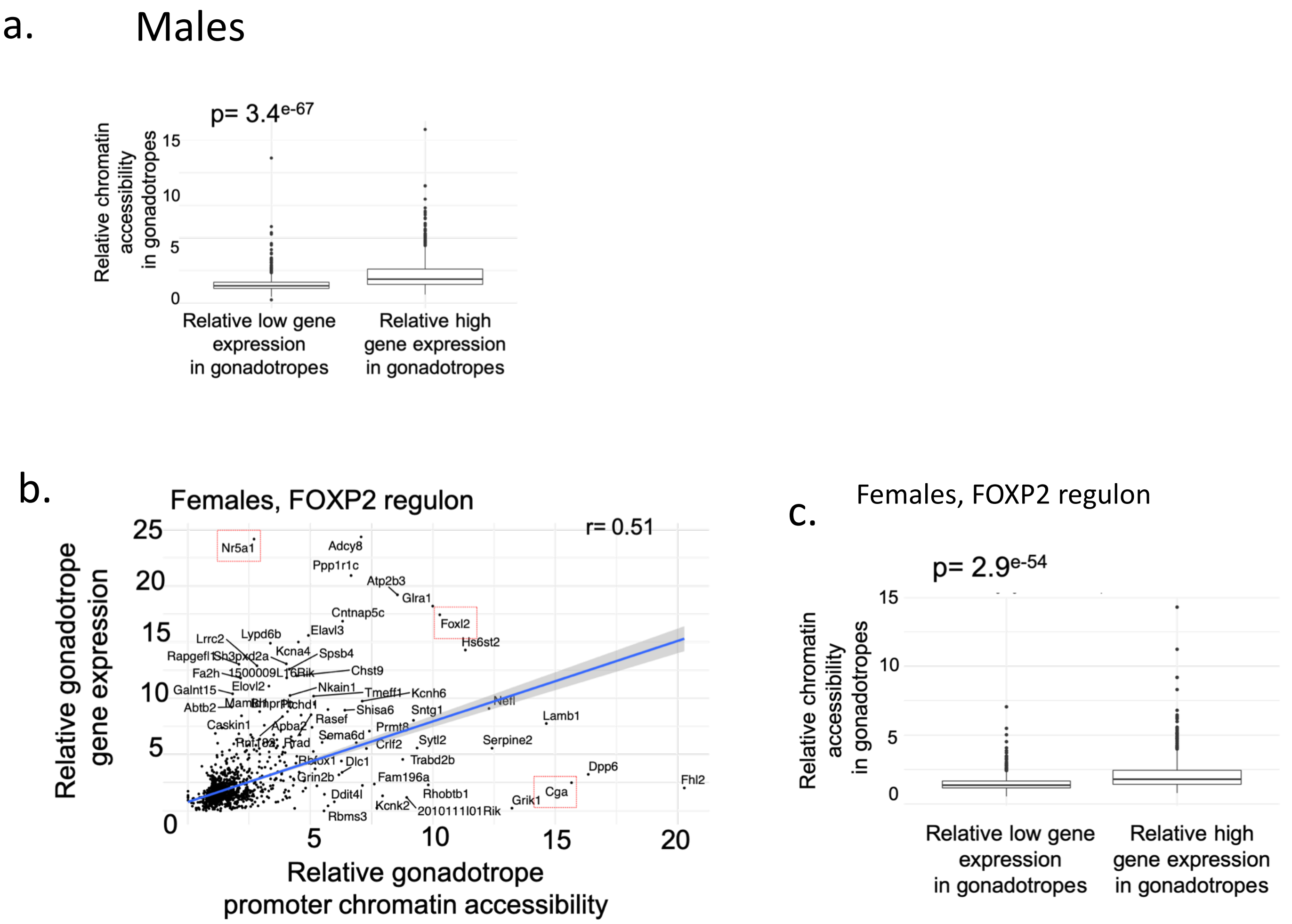

Supplementary Figure 24:
a. Boxplots of the relative chromatin accessibility within gene bodies for the 1676 genes composing the FOXP2 regulon separated into relatively low expression vs. relatively high expression in female (right) gonadotropes (consensus from 3 individual female samples). Two-sided Wilcoxon rank-sum tests (W = 158752). Left (low): min 0.22, max 11.13, lower whisker 0.46, lower box 1.10, median 1.30, upper box 1.58, upper whisker 2.30. Right (high): min 0.63, max 13.33, lower whisker 0.63, lower box 1.41, median 1.81, upper box 2.59, upper whisker 4.30. **b**. Scatter plot and correlation of the 1676 genes composing the FOXP2 regulon based on their relative gene expression and relative TSS chromatin accessibility in female gonadotropes (consensus from 3 individual female samples). Boxed in red are genes that are related to gonadotrope regulation. **c.** Boxplots of the relative chromatin accessibility within gene bodies for the FOXP2 regulon separated into relatively low expression vs. relatively high expression in female gonadotropes. Two-sided Wilcoxon rank-sum tests (W = 182337). Left (low): min 0.55, max 7.06, lower whisker 0.55, lower box 1.17, median 1.37, upper box 1.67, upper whisker 2.43. Right (high): min 0.80, max 14.32, lower whisker 0.80, lower box 1.43, median 1.80, upper box 2.45, upper whisker 3.96 Refer to **Fig. 8** for analysis of the FOXP2 regulon in males.

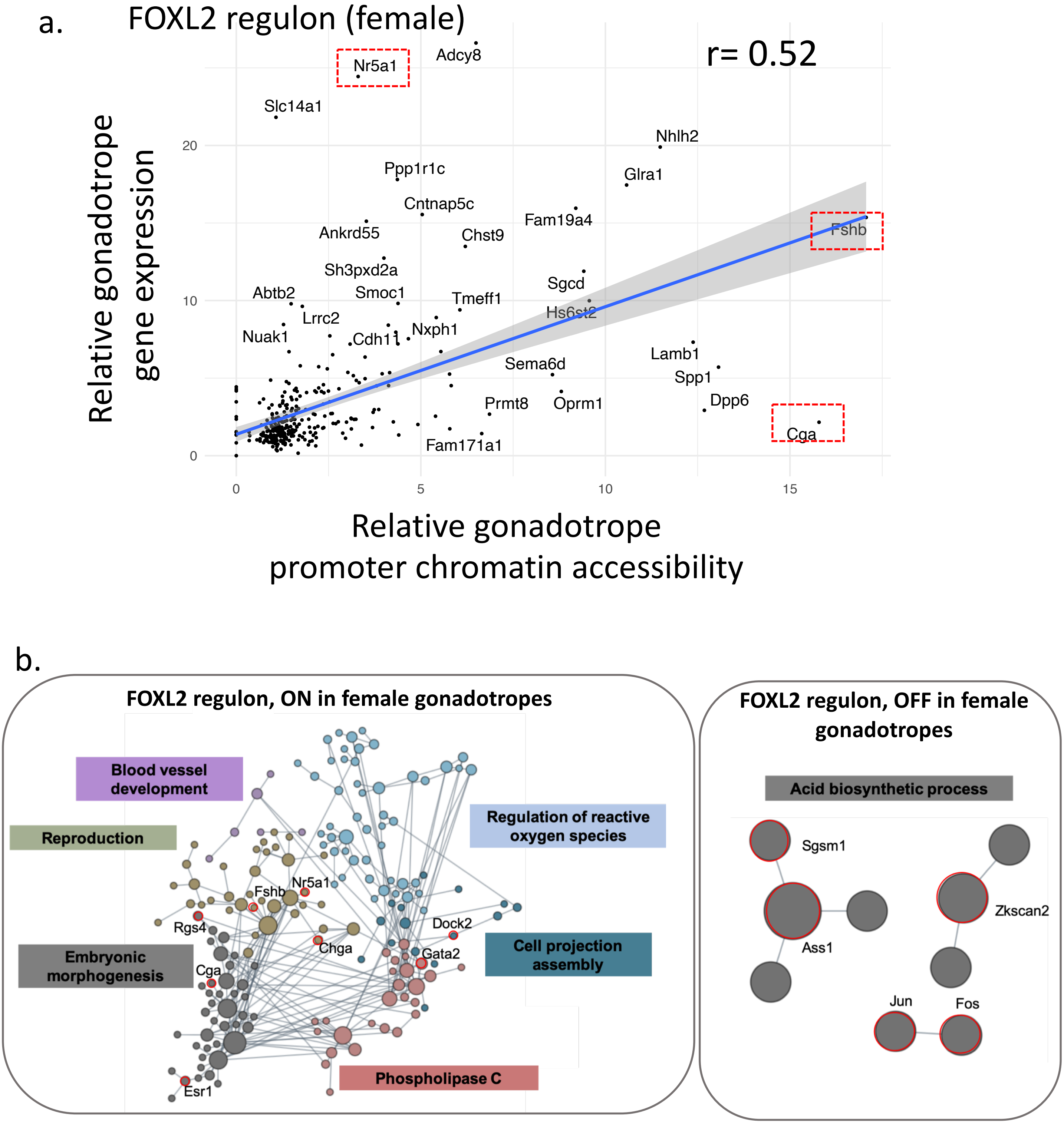

Supplementary Figure 25:

a. Scatter plot and correlation of the 331 genes composing the FOXL2 regulon based on their relative gene expression and relative TSS chromatin accessibility in female gonadotropes (consensus female from 3 individual female samples). Boxed in red are genes that are related to gonadotrope regulation. **b**. Gene network for the FOXL2 regulon targets that are ON in female gonadotropes (Left panel), and regulon targets that are OFF in female gonadotropes (Right panel; consensus female from 3 individual female samples).

FOXL2 regulon, gonadotropes

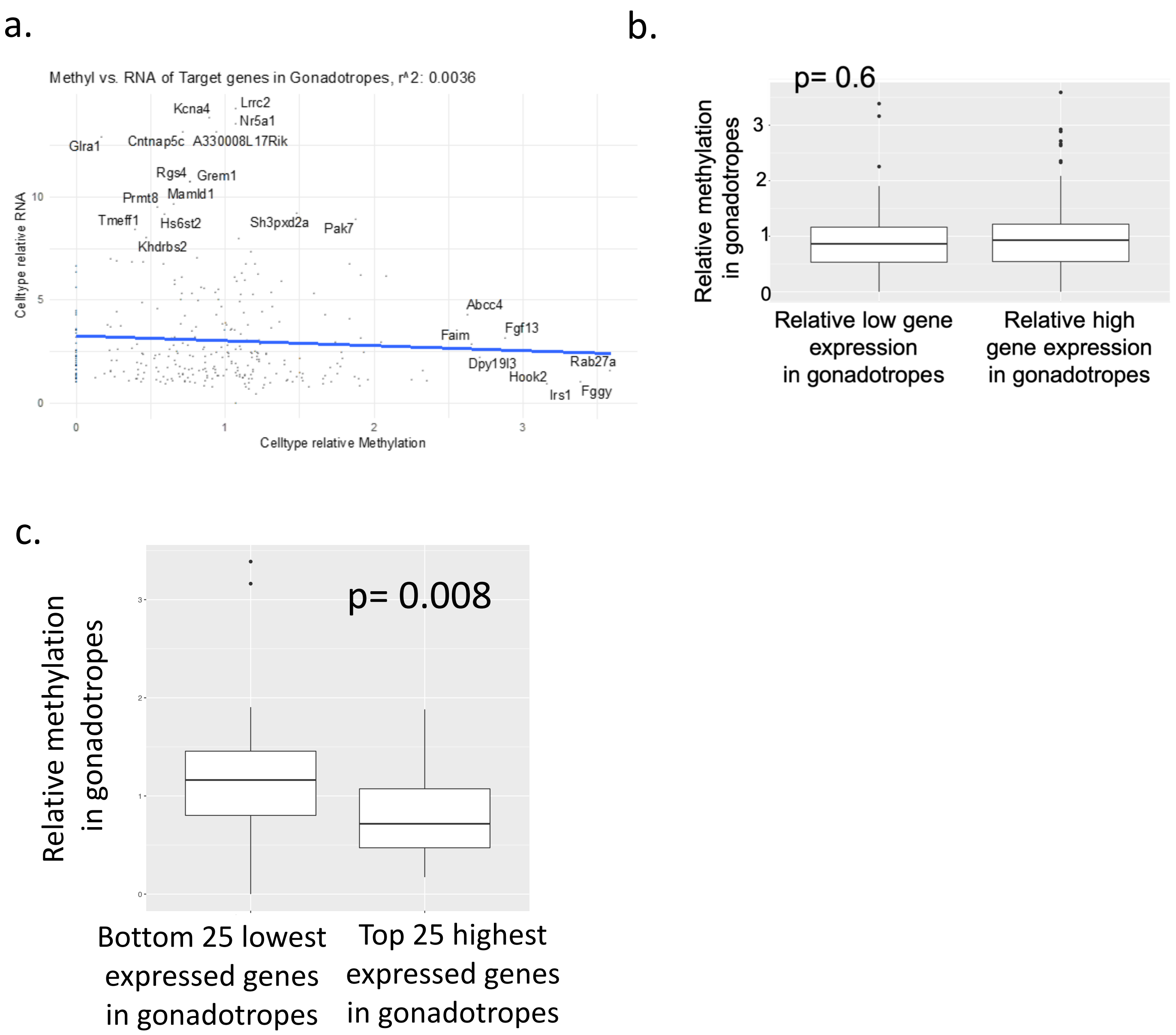

Supplementary Figure 26:
a. Scatter plot and correlation of the 331 genes composing the FOXL2 regulon based on their relative gene expression and relative methylation in male gonadotropes (consensus male from 3 individual male samples). **b**. Boxplots of relative methylation at the TSS for all 331 genes composing the FOXL2 regulon, separated into relatively low expression vs. relatively high expression in male gonadotropes (consensus male from 3 individual male samples). Two-sided Wilcoxon rank-sum tests (W = 8995). Left (low): min 0.00 max 3.39, lower whisker 0.00, lower box 0.53, median 0.86, upper box 1.16, upper whisker 1.90. Right (high): min 0.00, max 3.59, lower whisker 0.00, lower box 0.54, median 0.93, upper box 1.21, upper whisker 2.09. **c**. Boxplots of the relative methylation at the TSS for the 25 lowest and 25 highest expressed genes of the FOXL2 regulon in male gonadotropes (consensus male from 3 individual male samples). Two-sided Wilcoxon rank-sum tests (W = 175). Left (low): min 0.00, max 3.39, lower whisker 0.00, lower box 0.80, median 1.16, upper box 1.46, upper whisker 1.90. Right (high): min 0.17, max 1.88, lower whisker 0.17, lower box 0.47, median 0.72, upper box 1.07, upper whisker 1.88.

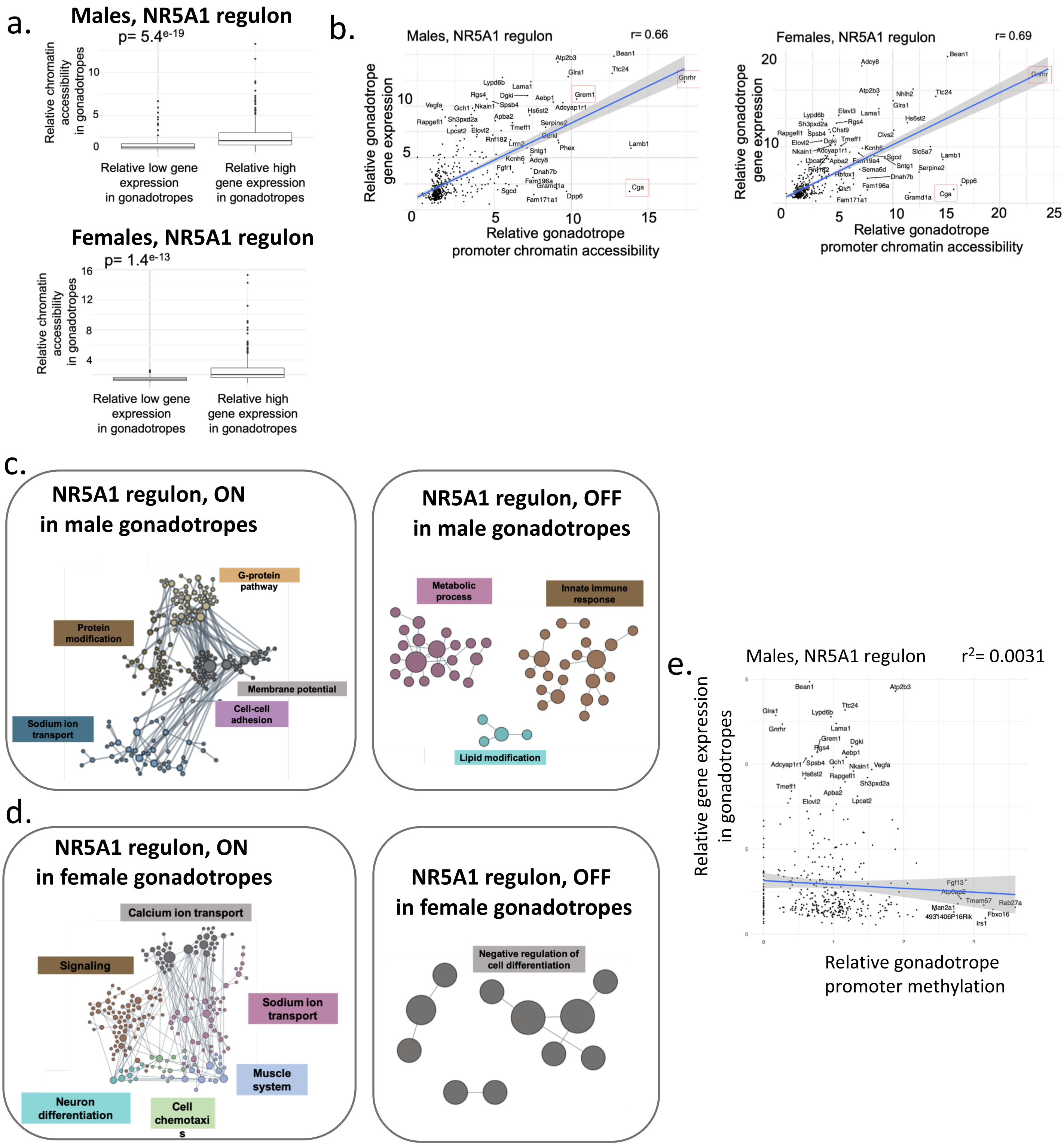

Supplementary Figure 27:

a. Boxplots of the relative chromatin accessibility within gene bodies for all 412 genes composing the NR5A1 regulon separated into relatively low expression vs. relatively high expression in male (Top panel) and female (Bottom panel) gonadotropes. Upper panel, Males: Two-sided Wilcoxon rank-sum tests (W = 6771). Left (low): min 0.83, max 6.69, lower whisker 0.83, lower box 1.15, median 1.35, upper box 1.74, upper whisker 2.45. Right (high): min 0.81, max 13.33, lower whisker 0.81, lower box 1.59, median 2.09, upper box 3.02, upper whisker 5.05. Lower panel, Females: Two-sided Wilcoxon rank-sum tests (W = 4419). Left (low): min 1.04, max 2.63, lower whisker 1.04, lower box 1.26, median 1.45, upper box 1.69, upper whisker 2.12. Right (high): min 0.94, max 15.36, lower whisker 0.94, lower box 1.64, median 2.04, upper box 2.94, upper whisker 4.85. **b**. Scatter plot and correlation of the 412 genes composing the NR5A1 regulon based on their relative gene expression and relative TSS chromatin accessibility in male (Left panel) and female (Right panel) gonadotropes. Boxed in red are genes that are related to gonadotrope regulation. **c**. Gene network for the NR5A1 regulon targets that are ON (Left panel), and regulon targets that are OFF in male gonadotropes (Right panel). **d**. Gene network for the NR5A1 regulon targets that are ON (Left panel), and regulon targets that are OFF in female gonadotropes (Right panel). e. Scatter plot and correlation of the 412 genes composing the NR5A1 regulon based on their relative gene expression and relative methylation in male gonadotropes. All panels are consensus from 3 animals.

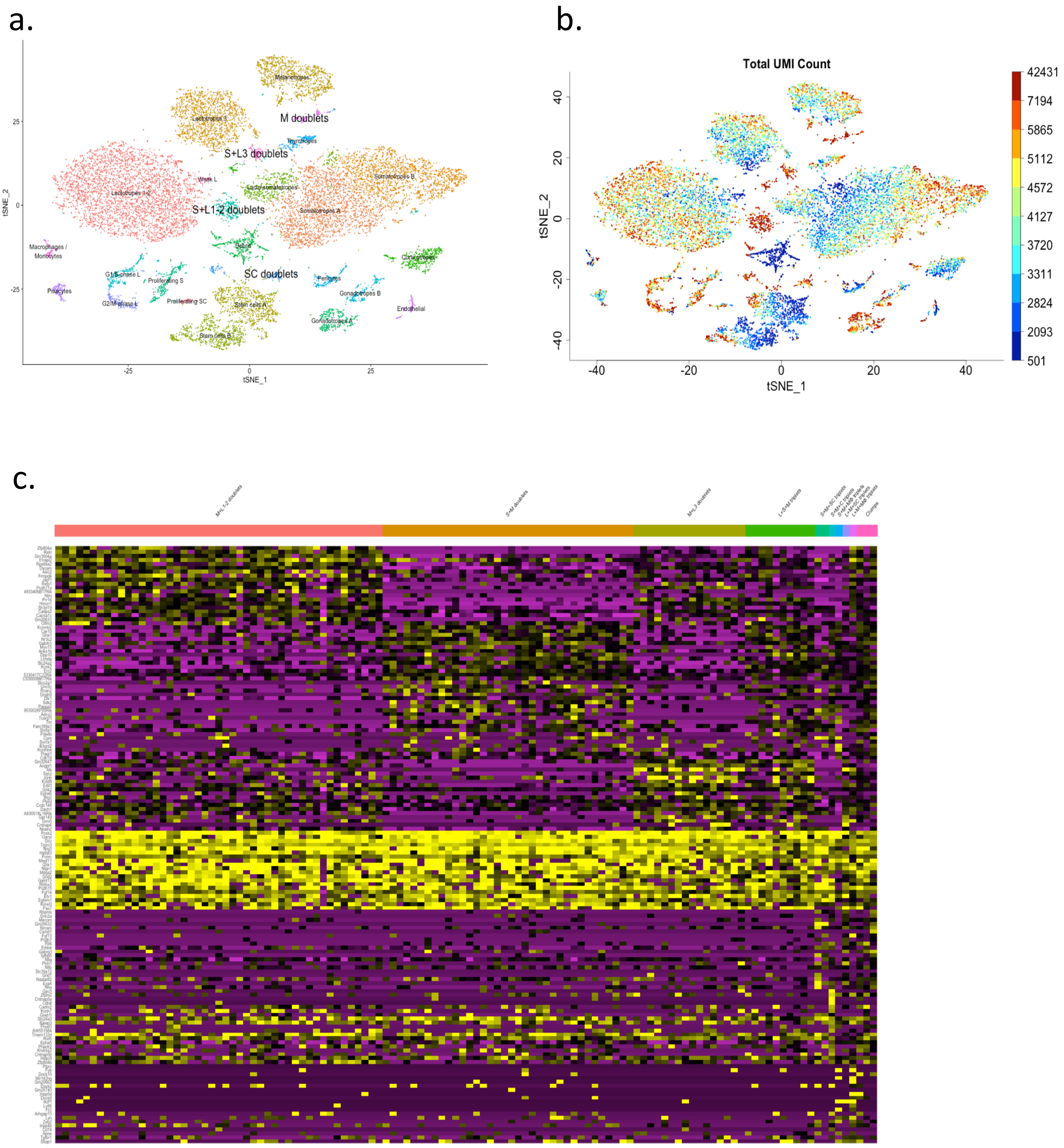

Supplementary Figure 28:
Doublet identification. **a**. Initial clustering with four doublet clusters highlighted. We used the main markers of each cluster to annotate them. Doublet clusters were identified as such because they display gene expression programs of two main clusters simultaneously. **b**. t-SNE plot color-coded by total UMI count. Doublet clusters consist mainly of barcodes with UMI counts in the highest 10th quantile, and, on average, twice as high as the median. **c**. heatmap of gene expression of the top-20 markers of a subset of main cell types for barcodes classified as Melanotrope doublets in our clustering analysis. Melanotropes doublets can be seen simultaneously expressing the melanotrope gene program as well as at least one other gene program. The doublet (multiplet) cell type(s) can be inferred based on which gene programs are expressed.

a.

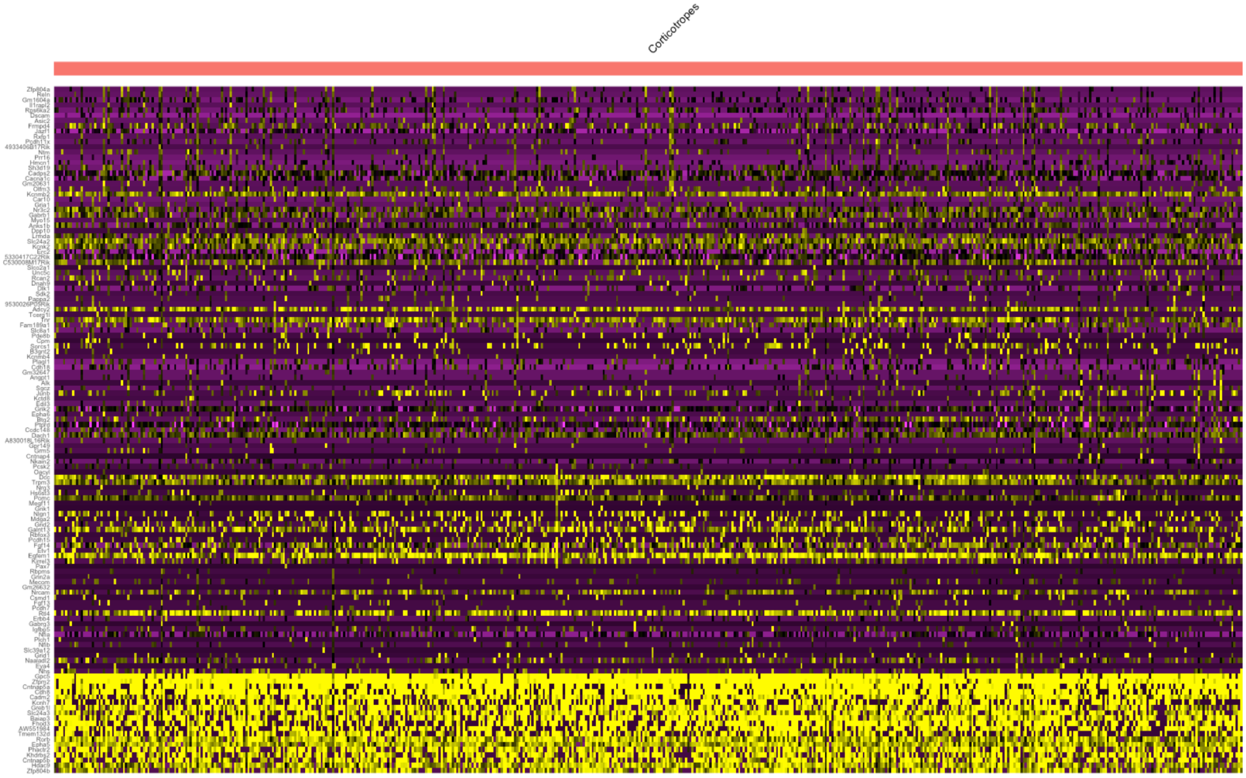

b.

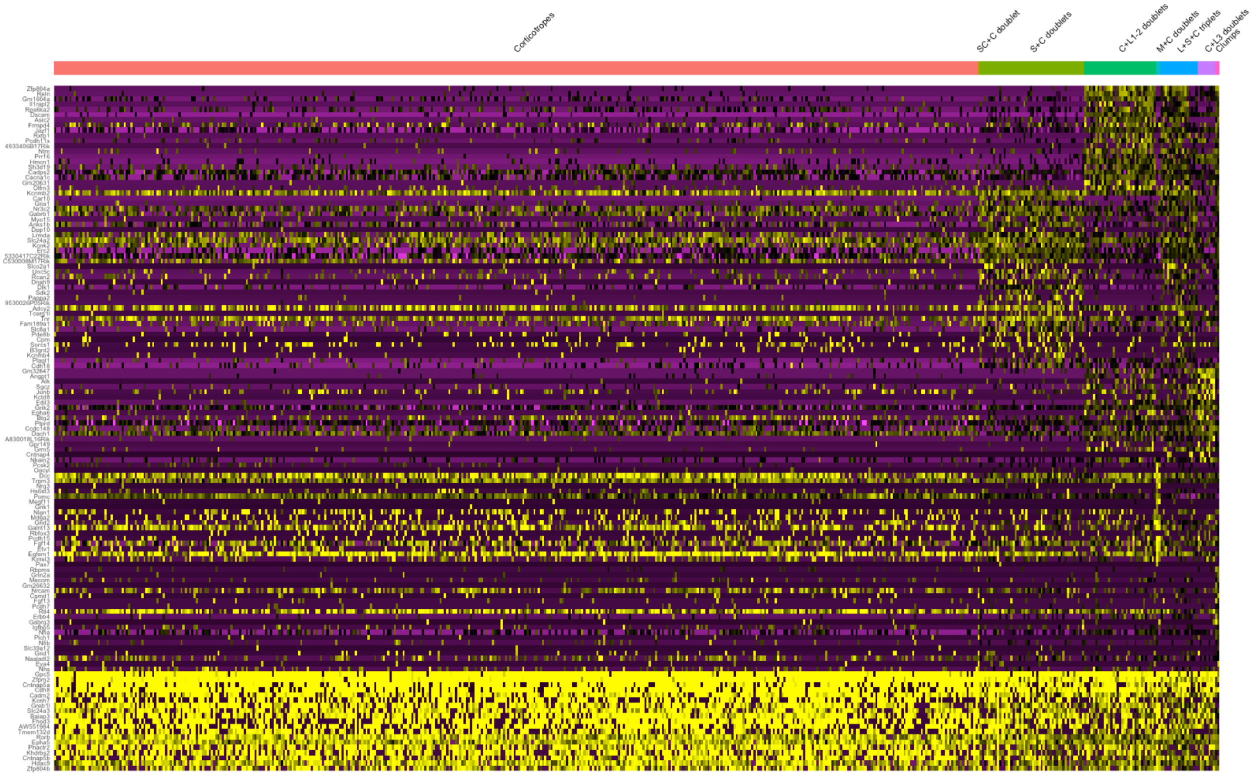

Supplementary Figure 29:
a. Heatmap of the Corticotropes cluster after our intial clustering analysis. Many doublets, represented by vertical streaks, which are gene expression from a different cell type, can be observed. b. Heatmap of the initial Corticotropes cluster after doublet/multiplet identification. The Corticotropes are now clean of other cell type contamination.

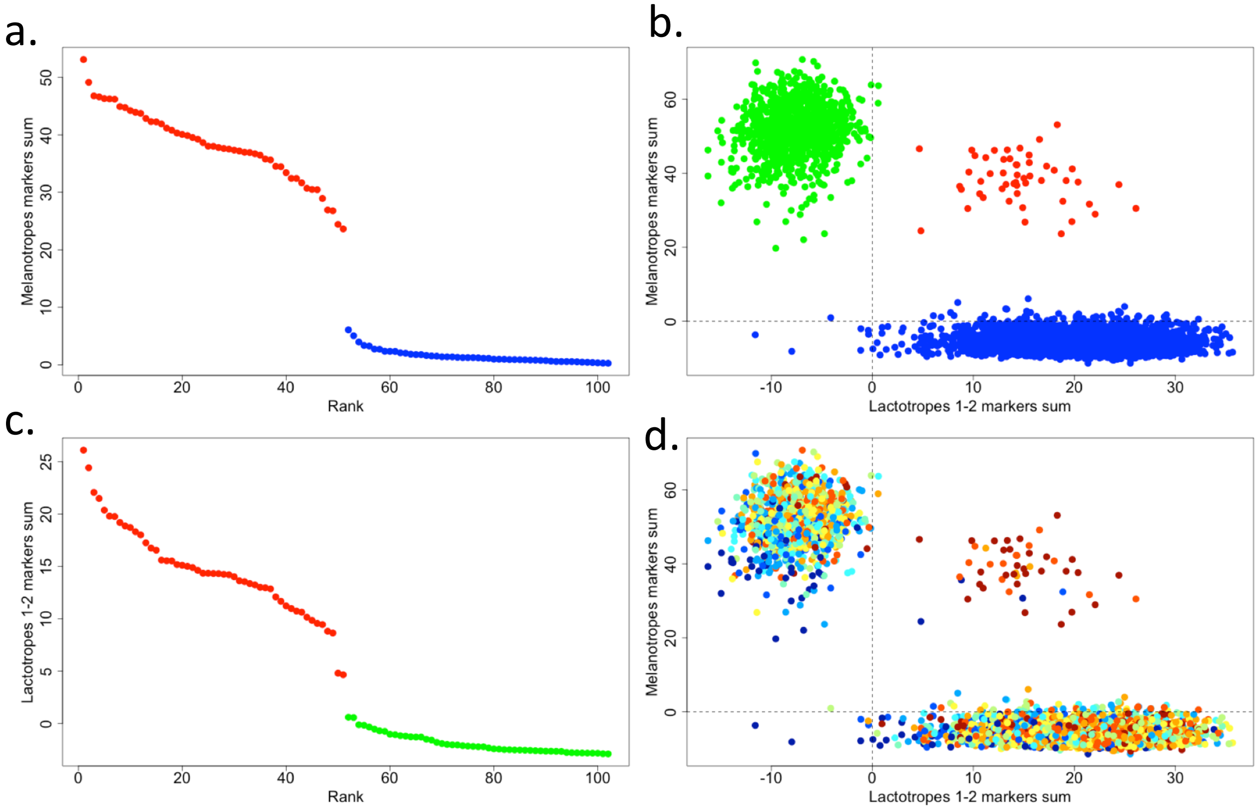

Supplementary Figure 30:
Example of verification plots for doublet identities; here Melanotropes - Lactotropes1-2 doublets. a. Rank plot of M+L1-2 doublets (red) and Lactotropes 1-2 (blue) ranked by the sum of their (scaled) expression of melanotrope markers. M+L1-2 doublets break from the rest of lactotropes. b. Rank plot of M+L1-2 doublets (red) and Melanotropes (green) ranked by the sum of their (scaled) expression of lactotropes1-2 markers. c, d. Combined markers plots color-coded by cell identity (c) and UMI count (d).

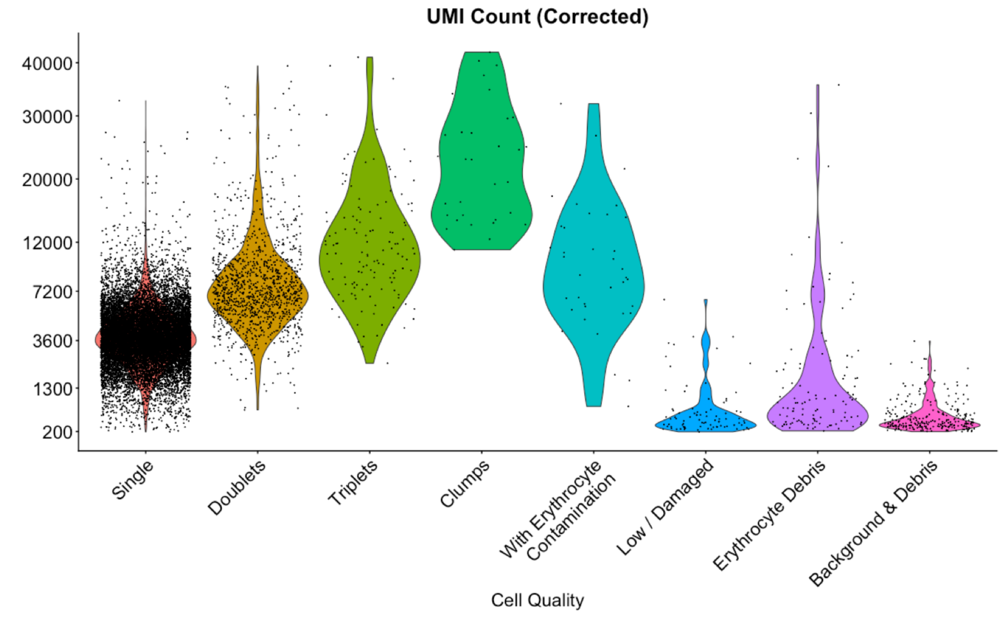

Supplementary Figure 31:
UMI Count distributions per cell quality and multiplet order in the merged females sample. UMI counts were corrected, on a per sample basis, for background (ambient RNA) by the mean UMI count in background barcodes (barcodes not called as cells in the CellRanger pipeline). A few background barcodes got passed the cell calling threshold, and thus appear in this figure. Only single-cell barcodes were retained for downstream analysis.

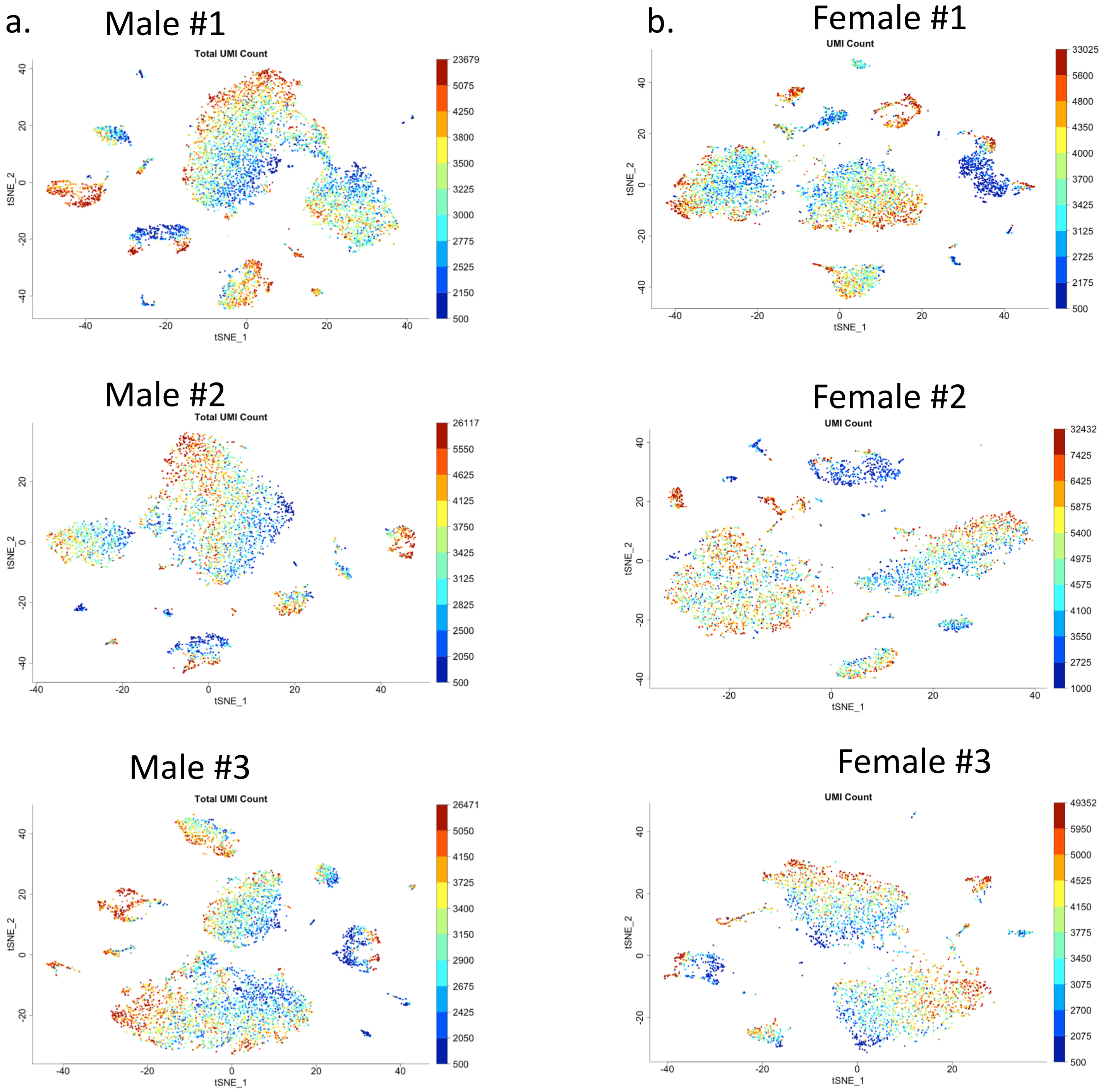

Supplementary Figure 32: UMI count individual snap-frozen pituitary samples

UMI counts are shown for each individual male (a) and female (b) samples after removal of doublets and debris.

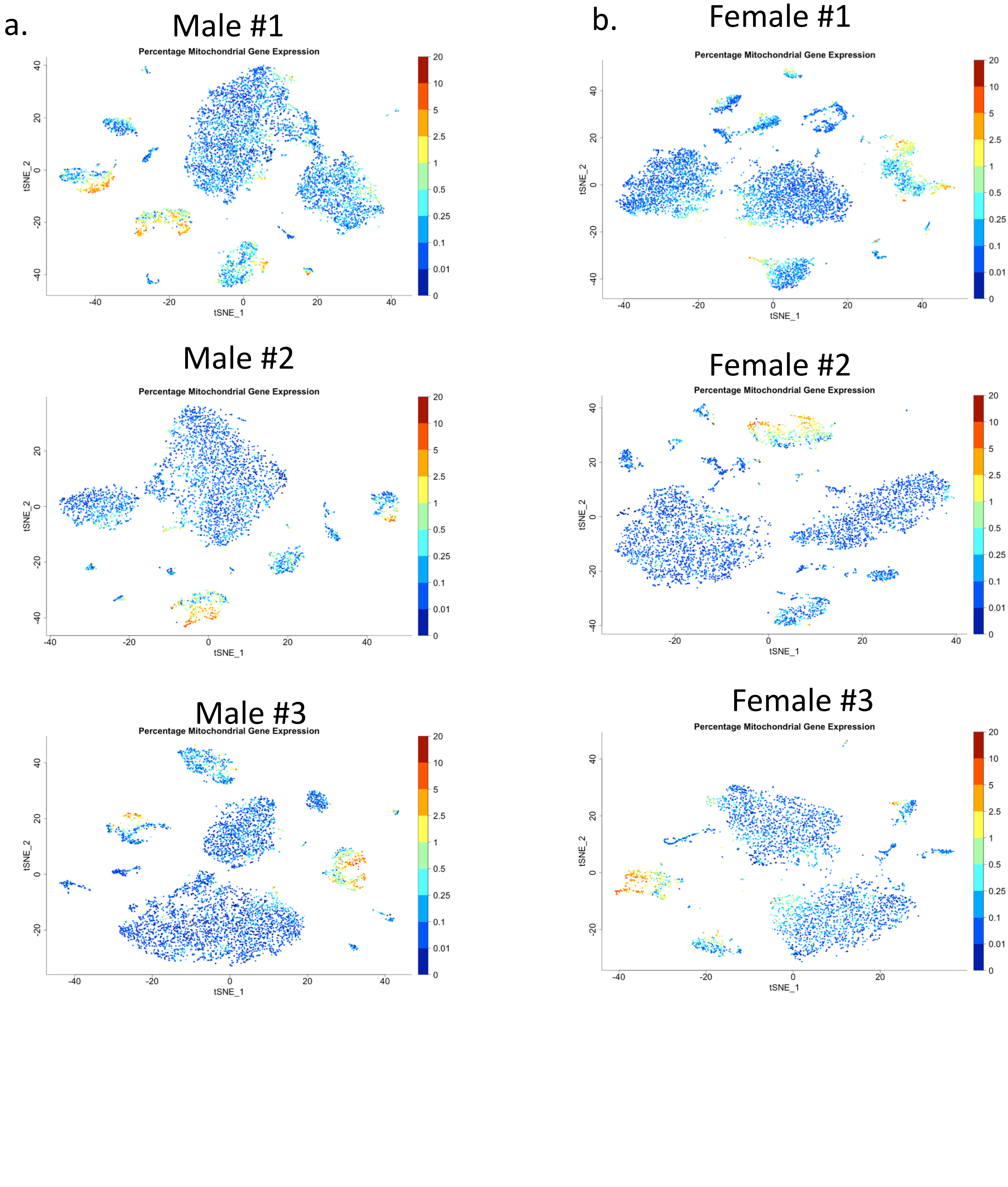

Supplementary Figure 33: Percentage of mitochondrial gene expression for individual snap-frozen pituitary samples

Mitochondrial gene expression t-SNE are shown for each individual male (a) and female (b) samples after removal of doublets and debris.

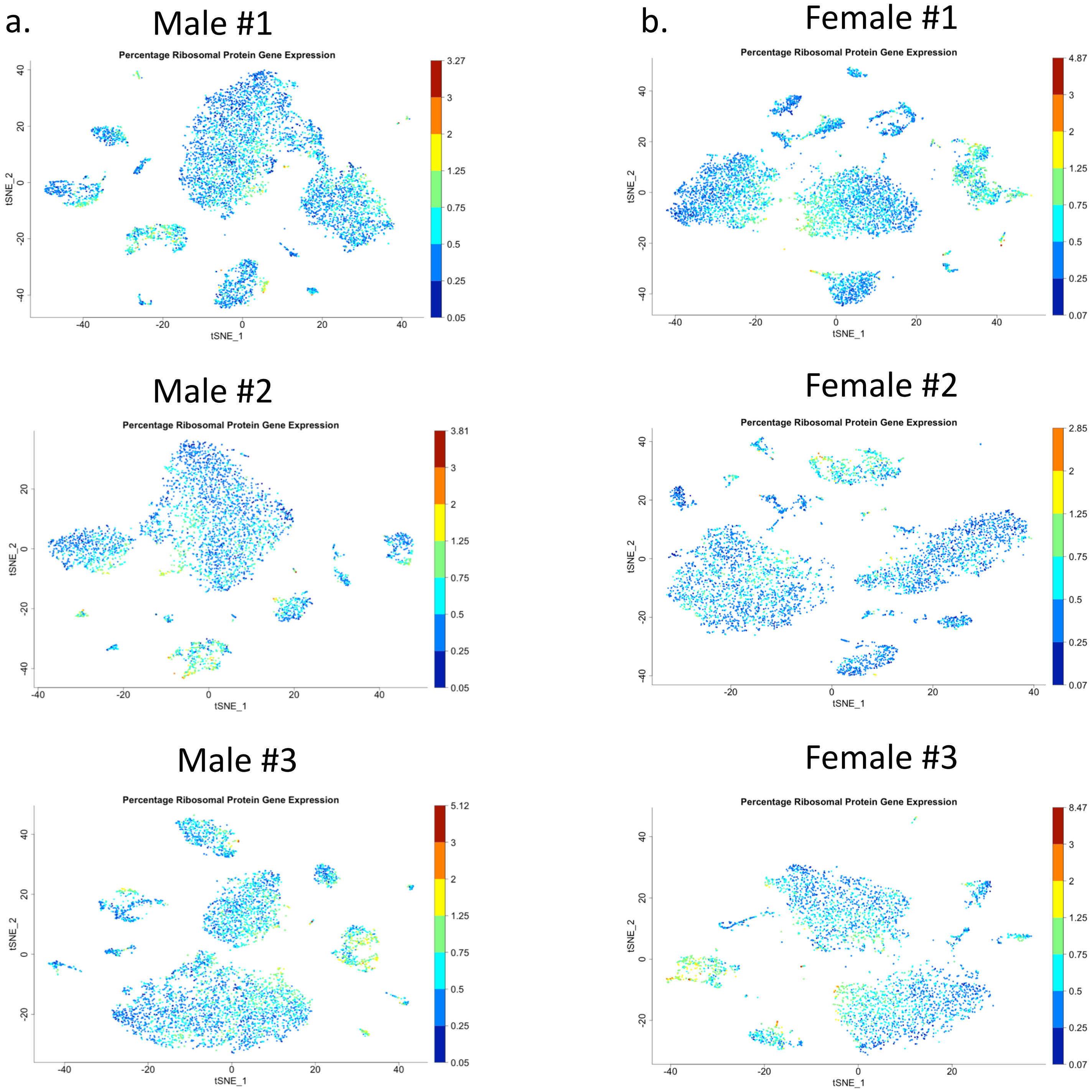

Supplementary Figure 34: Percentages of ribosomal gene expression for individual snap-frozen pituitary samples

Ribosomal gene expression t-SNE are shown for each individual male (a) and female (b) samples after removal of doublets and debris.
