# Supplementary material for: Single nucleus multi-omics regulatory landscape of the murine pituitary
Source: Nat Commun. 2021 May 11;12:2677. doi: 10.1038/s41467-021-22859-w (PMC8113460; doi:10.1038/s41467-021-22859-w)
Supplement: Supplementary file 3 — Reporting Summary [file 41467_2021_22859_MOESM3_ESM.pdf]

nature research

Corresponding author(s): Frederique Ruf-Zamojski and Stuart Sealfon

Last updated by author(s): Feb 19, 2021

Reporting Summary

Nature Research wishes to improve the reproducibility of the work that we publish. This form provides structure for consistency and transparency in reporting. For further information on Nature Research policies, see our Editorial Policies and the Editorial Policy Checklist.

Statistics

For all statistical analyses, confirm that the following items are present in the figure legend, table legend, main text, or Methods section.

n/a	Confirmed	
☐	☒	The exact sample size (*n*) for each experimental group/condition, given as a discrete number and unit of measurement
☐	☒	A statement on whether measurements were taken from distinct samples or whether the same sample was measured repeatedly
☐	☒	The statistical test(s) used AND whether they are one- or two-sided<br>*Only common tests should be described solely by name; describe more complex techniques in the Methods section.*
☐	☒	A description of all covariates tested
☐	☒	A description of any assumptions or corrections, such as tests of normality and adjustment for multiple comparisons
☐	☒	A full description of the statistical parameters including central tendency (e.g. means) or other basic estimates (e.g. regression coefficient) AND variation (e.g. standard deviation) or associated estimates of uncertainty (e.g. confidence intervals)
☐	☒	For null hypothesis testing, the test statistic (e.g. *F*, *t*, *r*) with confidence intervals, effect sizes, degrees of freedom and *P* value noted<br>*Give P values as exact values whenever suitable.*
☒	☐	For Bayesian analysis, information on the choice of priors and Markov chain Monte Carlo settings
☐	☒	For hierarchical and complex designs, identification of the appropriate level for tests and full reporting of outcomes
☐	☒	Estimates of effect sizes (e.g. Cohen's *d*, Pearson's *r*), indicating how they were calculated

Our web collection on statistics for biologists contains articles on many of the points above.

Software and code

Policy information about availability of computer code

Data collection	A methylation mapping pipeline (cemba-data.rtfd.io) was implemented for all the sn methylome-based technologies developed by the Ecker Lab and described in the Methods.<br>Single nuclei RNA and single nuclei ATAC libraries were sequenced at the New York Genome Center, and Fastq files were created from Bcl files in Cell Ranger.
Data analysis	R was used for analysis, with specific packages including Seurat v3.1.1, Cell Ranger pipeline v3.0.2, Cell Ranger-ATAC pipeline version 1.2.0, Pathway Level Information ExtractoR framework (PLIER), SCENIC, and Cicero packages. Signac v. 0.1.5 was used to confirm clustering. The functional regulon modules were obtained from the HumanBase resource (https://hb.flatironinstitute.org/module). Conservation analysis were performed using the PhastCon and PhyloP(60) packages.

For manuscripts utilizing custom algorithms or software that are central to the research but not yet described in published literature, software must be made available to editors and reviewers. We strongly encourage code deposition in a community repository (e.g. GitHub). See the Nature Research guidelines for submitting code & software for further information.

Data

Policy information about availability of data

All manuscripts must include a data availability statement. This statement should provide the following information, where applicable:

- Accession codes, unique identifiers, or web links for publicly available datasets
- A list of figures that have associated raw data
- A description of any restrictions on data availability

he datasets (scRNAseq, snRNAseq, snATACseq) and sn methylation data generated in the present study are deposited in GEO. The sn mouse pituitary multi-omics atlas can be browsed via a web-based portal accessible at snpituitaryatlas.princeton.edu.

Field-specific reporting

Please select the one below that is the best fit for your research. If you are not sure, read the appropriate sections before making your selection.

☒ Life sciences ☐ Behavioural & social sciences ☐ Ecological, evolutionary & environmental sciences

For a reference copy of the document with all sections, see nature.com/documents/nr-reporting-summary-flat.pdf

Life sciences study design

All studies must disclose on these points even when the disclosure is negative.

Sample size	We performed single nuclei analysis on 3 male and 3 female murine samples. We also processed samples for single cell RNAseq on 5 male and 2 female pituitaries (Supplementary data).
Data exclusions	No data were excluded from analyses.
Replication	The data were reproducible. We performed single nuclei analysis on 3 male and 3 female murine samples. We also processed samples for single cell RNAseq on 5 male and 2 female pituitaries (Supplementary data).
Randomization	We did not do any randomization. For nuclei extraction, the 3 male samples were processed together on a single day, and the 3 female samples were processed together on another single day.
Blinding	We did not do any blinding. It was not relevant for the study.

Reporting for specific materials, systems and methods

We require information from authors about some types of materials, experimental systems and methods used in many studies. Here, indicate whether each material, system or method listed is relevant to your study. If you are not sure if a list item applies to your research, read the appropriate section before selecting a response.

Materials & experimental systems

n/a	Involved in the study	
☒	☐	Antibodies
☐	☒	Eukaryotic cell lines
☒	☐	Palaeontology and archaeology
☐	☒	Animals and other organisms
☒	☐	Human research participants
☒	☐	Clinical data
☒	☐	Dual use research of concern

Methods

n/a	Involved in the study	
☒	☐	ChIP-seq
☒	☐	Flow cytometry
☒	☐	MRI-based neuroimaging

Eukaryotic cell lines

Policy information about cell lines

Cell line source(s)	The parent LbT2 cell line was given as a gift by Dr Pamela Mellon (UCSD) and obtained under an MTA.
Authentication	We confirmed expression of Fshb, Lhb and Cga from the mouse pituitary cell line. The expression of these genes are gonadotrope specific, confirming the source of the cells. The cells have now been authenticated by STR fingerprinting.
Mycoplasma contamination	The cells were not tested for mycoplasma at the time of the experiments.
Commonly misidentified lines (See ICLAC register)	N/A

Animals and other organisms

Policy information about studies involving animals; ARRIVE guidelines recommended for reporting animal research

Laboratory animals	Male and randomly-cycling C57BL/6 female mice aged 10-12 weeks. Animals were on a 12-hour on, 12-hour off light cycle (lights on at 7 AM; off at 7 PM).
Wild animals	N/A

Field-collected samples	N/A
Ethics oversight	All murine work was conducted at McGill University (Montreal, Quebec, Canada) under animal use protocol 5204, as approved by the Facility Animal Care Committee of the Goodman Cancer Research Centre.

Note that full information on the approval of the study protocol must also be provided in the manuscript.
